# Repression of mRNA translation initiation by GIGYF1 via disrupting the eIF3-eIF4G1 interaction

**DOI:** 10.1126/sciadv.adl5638

**Published:** 2024-07-17

**Authors:** Jung-Hyun Choi, Jun Luo, Geoffrey G. Hesketh, Shuyue Guo, Angelos Pistofidis, Reese Jalal Ladak, Yuxin An, Parisa Naeli, Tommy Alain, T. Martin Schmeing, Anne-Claude Gingras, Thomas Duchaine, Xu Zhang, Nahum Sonenberg, Seyed Mehdi Jafarnejad

**Affiliations:** ^1^ Rosalind and Morris Goodman Cancer Institute, McGill University, Montreal, QC H3A 1A3, Canada.; ^2^ Department of Biochemistry, McGill University, Montreal, QC H3A 1A3, Canada.; ^3^ Department of Biochemistry & Molecular Biology, Dalhousie University, Halifax, NS, Canada.; ^4^ Patrick G. Johnston Centre for Cancer Research, Queen’s University Belfast, Belfast BT9 7AE, UK.; ^5^ Department of Biochemistry, Microbiology and Immunology, Children's Hospital of Eastern Ontario Research Institute, University of Ottawa, Ottawa, ON, Canada.; ^6^ Centre for Systems Biology, Lunenfeld-Tanenbaum Research Institute, Sinai Health System, Toronto, ON M5G 1X5, Canada.; ^7^ Department of Molecular Genetics, University of Toronto, Toronto, ON M5S 1A1, Canada.

## Abstract

Viruses can selectively repress the translation of mRNAs involved in the antiviral response. RNA viruses exploit the Grb10-interacting GYF (glycine-tyrosine-phenylalanine) proteins 2 (GIGYF2) and eukaryotic translation initiation factor 4E (eIF4E) homologous protein 4EHP to selectively repress the translation of transcripts such as *Ifnb1*, which encodes the antiviral cytokine interferon-β (IFN-β). Herein, we reveal that GIGYF1, a paralog of GIGYF2, robustly represses cellular mRNA translation through a distinct 4EHP-independent mechanism. Upon recruitment to a target mRNA, GIGYF1 binds to subunits of eukaryotic translation initiation factor 3 (eIF3) at the eIF3-eIF4G1 interaction interface. This interaction disrupts the eIF3 binding to eIF4G1, resulting in transcript-specific translational repression. Depletion of GIGYF1 induces a robust immune response by derepressing IFN-β production. Our study highlights a unique mechanism of translational regulation by GIGYF1 that involves sequestering eIF3 and abrogating its binding to eIF4G1. This mechanism has profound implications for the host response to viral infections.

## Teaser

GIGYF1 represses translation of target mRNAs by disrupting the eIF3-eIF4G1 interaction in the preinitiation complex.

## INTRODUCTION

In eukaryotes, cap-dependent mRNA translation initiation involves the initial recognition of the 5′ m^7^G cap structure by a multiprotein complex known as eIF4F, which consists of the cap-binding protein eukaryotic translation initiation factor 4E (eIF4E), the RNA helicase eIF4A, and the scaffold protein eIF4G1. Following cap recognition, the large eIF3 complex bridges eIF4G1 and the preassembled preinitiation complex (PIC), which includes the 40*S* ribosomal subunit, eIF1, eIF1A, and eIF2-GTP-Met-tRNAi (*1*). PIC scans along the 5′ untranslated region (5′UTR) of the mRNA until it reaches the translation start codon (typically AUG). This leads to the recruitment of the 60*S* ribosomal subunit and the subsequent assembly and precise positioning of the 80*S* ribosome at the start codon, thus the commencement of the polypeptide synthesis (*1*).

In mammals, the eIF3 complex consists of 13 subunits (eIF3A-J). The core function of eIF3 in recruiting the PIC to the mRNA exhibits a high degree of conservation from budding yeast to higher eukaryotes. Nevertheless, the *Saccharomyces cerevisiae* eIF3 complex, composed of five subunits (eIF3a, eIF3b, eIF3c, eIF3g, and eIF3i), is considerably smaller than the 13-subunit mammalian eIF3 complex (*2*). While eIF3 plays prominent roles in various steps of mRNA translation, knockdown studies in human cells suggested that some eIF3 subunits are dispensable for general translation initiation (*3*). This suggests that certain noncore subunits of mammalian eIF3 may have evolved to fulfill specialized regulatory functions. Several studies have documented both positive and negative impacts of individual eIF3 subunits on the translation of specific mRNAs (*4*–*7*). For example, the eIF3D and eIF3L subunits directly bind to the 5′ m^7^G cap and regulate the cap-dependent translation initiation of specific mRNAs (*8*, *9*). Nonetheless, our mechanistic understanding of how eIF3 regulates the translation of specific mRNAs and its potential role in the regulation of transcript-specific translation by other factors such as RNA-binding proteins (RBPs) and microRNAs remains limited.

The Grb10-interacting GYF (glycine-tyrosine-phenylalanine) proteins 1 and 2 (GIGYF1 and GIGYF2, also known as PERQ1 and PERQ2) were originally identified through a yeast two-hybrid screen for their interaction with the adaptor protein Grb10 (*10*). The GYF domain in the mid-region of GIGYF1/2 adopts a structurally conserved fold shared among a variety of proteins across diverse eukaryotic species. The GYF domain mediates interactions with proline-rich sequences (PRSs) (*11*), allowing GIGYF1/2 to engage with several RBPs that have one or more PRSs, including tristetraprolin (TTP), zinc finger protein 598 (ZNF598), and the trinucleotide repeat-containing gene 6 (TNRC6A, TNRC6B, and TNRC6C) proteins that are among the core components of the microRNA-induced silencing complex (miRISC). Thereby, through interactions with these RBPs, GIGYF1/2 can be recruited to specific mRNAs. GIGYF1/2 proteins also harbor a conserved N-terminal motif (YXYXXXXLΦ), where Φ represents a hydrophobic amino acid, which enables their specific interactions with the eIF4E homologous protein, 4EHP (also known as eIF4E2), but not to eIF4E (*12*, *13*). Similar to eIF4E, 4EHP binds to m^7^G cap, albeit with lower efficiency. Yet, 4EHP does not interact with eIF4G and thereby fails to initiate the canonical cap-dependent translation initiation (*14*). Thus, through binding to 4EHP, GIGYF1/2 could repress the translation of specific mRNAs to which they are recruited via their GYF:PRS-mediated interacting RBPs (e.g., TTP). However, formation of the repressor complex with 4EHP accounts for only a fraction of translational repression of the target mRNAs by GIGYF2 (*13*, *15*, *16*), highlighting the existence of an additional, 4EHP-independent mechanism of translational repression by GIGYF2. Despite several investigations into the mechanism of GIGYF2-mediated translational regulation (*12*, *15*, *17*–*21*), the functional mechanism and biological significance of GIGYF1 remain poorly defined.

Type I interferons (IFN-α and IFN-β) play crucial roles in the innate antiviral immune system (*22*), initiating a signaling cascade via the IFN-α/β receptor (IFNAR) that triggers the Janus kinase–signal transducers and activators of transcription (STAT) pathway and promotes expression of IFN-stimulated genes (ISGs) with direct antiviral effects (*23*). However, excessive levels of IFNs can lead to autoinflammatory and autoimmune disorders (*24*–*26*). Consequently, maintenance of immune homeostasis necessitates a finely tuned regulatory mechanism capable of rapid production of IFNs upon detection of viral infection while prohibiting “overshooting” of the immune response. In this context, translational regulation emerges as a rapid and flexible means of controlling IFN production in response to viral infections (*27*). We recently reported the involvement of the GIGYF2/4EHP complex in repressing the translation of *Ifnb1* mRNA and the subsequent restraint of IFN-β production during RNA virus infections (*16*, *28*). We and others also reported that the nonstructural protein 2 (NSP2) encoded by severe acute respiratory syndrome coronavirus 2 (SARS-CoV-2) interacts with the GIGYF2/4EHP complex (*16*, *29*, *30*). We showed that this interaction enhances the GIGYF2/4EHP-mediated translational repression of *Ifnb1* mRNA, to dampen the antiviral immune response (*16*). Although GIGYF1 also interacts with 4EHP in a similar manner to GIGYF2, no discernible interaction between NSP2 and GIGYF1 has been observed (*16*, *30*, *31*). These findings indicate the potential existence of distinct functional and biological roles for GIGYF1 and GIGYF2 proteins, which remain unexplored.

Here, we report a previously unknown mechanism of regulation of mRNA translation by GIGYF1. Our findings show that upon recruitment to an mRNA, GIGYF1 binds to subunits of eIF3 at the interface of eIF3-eIF4G interaction, abrogates the interaction between PIC and eIF4F complex, and consequently represses mRNA translation initiation. In addition, we demonstrate that this mechanism has a profound impact on the host cell response to RNA virus infection and that depletion of GIGYF1 leads to a strong immune response due to derepression of *Ifnb1* mRNA translation.

## RESULTS

### GIGYF1 potently represses IFN-β production in a 4EHP-independent manner

**Fig. 1.**
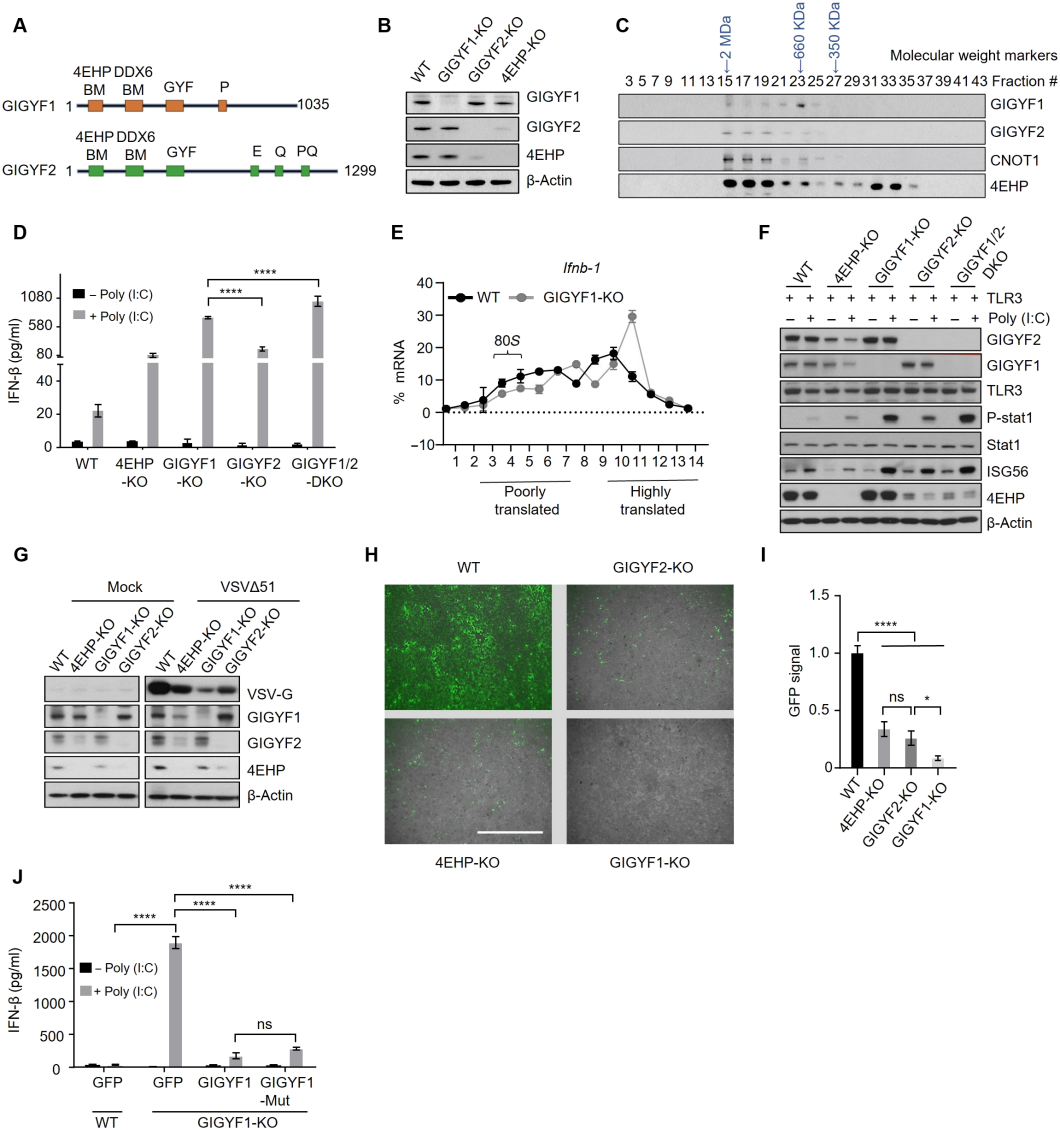
GIGYF1 enables RNA virus replication by repressing the translation of *Ifnb1* mRNA through a 4EHP-independent mechanism. (**A**) Schematic representation of the critical motifs in human GIGYF1 and GIGYF2 proteins. Domain sizes are not depicted at scale. Images were generated with BioRender.com. (**B**) WB analysis with the indicated antibodies of WT, GIGYF1-KO, GIGYF2-KO, and 4EHP-KO HEK293 cell lysates. (**C**) Fractionation of endogenous proteins by size exclusion chromatography, followed by WB with the indicated antibodies. Elution position of the molecular weight markers is shown. (**D**) ELISA measurement of IFN-β production in TLR3-HEK293 cells with indicated genotypes following 6 hours of poly(I:C) (1 μg/ml) stimulation. (**E**) Polysome profiling and quantitative reverse transcription polymerase chain reaction (qRT-PCR) analysis of *Ifnb1* mRNA translation in poly(I:C)-treated WT and GIGYF1-KO HEK293 cells. 80*S* peak corresponds to fractions 4 to 5. An increase in the fraction number (*x* axis) corresponds with higher translation rate. (**F**) WB analysis of cell lysates from (D). (**G**) WB analysis of viral protein VSV-G in the HEK293 cells with indicated genotypes 12 hours after infection with mock or VSVΔ51. (**H** and **I**) Virus protection assay for measurement of the impact of GIGYF1-KO on viral replication and spread. HEK293 cells with indicated genotypes were treated with poly(I:C) (1 μg/ml) for 6 hours. Culture media from the treated cells were transferred to recipient untreated HEK293 cells, which were subsequently exposed to the GFP-expressing VSVΔ51-GFP (multiplicity of infection = 0.01). Virus replication was visualized by fluorescence microscopy. Scale bar, 1 mm (H) and the GFP signal was quantified (I). (**J**) GFP control, v5-GIGYF1, or the 4EHP-binding mutant (Y39A, Y41A, M46A, L47A) v5-GIGYF1-Mut plasmids were transfected into the WT or GIGYF1-KO cells. IFN-β ELISA was performed following 6 hours of poly(I:C) (1 μg/ml) stimulation. Data are presented as mean ± SD (*n* = 3). ns, nonsignificant, *P < 0.05, ****P < 0.0001; one-way analysis of variance (ANOVA) with Bonferroni’s post hoc test.

GIGYF1 and GIGYF2 exhibit a high degree (55%) of similarity in their amino acid sequences, particularly in their N-terminal and central regions that respectively contain the critical 4EHP-binding (YXYXXXXLΦ), DDX6-binding, and GYF motifs (Fig. 1A and fig. S1). The two proteins, however, substantially diverge in the sequence of their C-terminal regions, with GIGYF1 containing an exclusive poly-proline (PolyP) sequence and GIGYF2 featuring stretches of glutamic acid–rich (E) and glutamine (Q) or proline and glutamine (PQ)–rich sequences (Fig. 1A and fig. S1). While GIGYF2 and 4EHP costabilize each other, as previously reported (*12*) and indicated by the destabilization of both proteins upon CRISPR-mediated knockout (KO) of either GIGYF2 or 4EHP, depletion of GIGYF1 had no tangible impact on 4EHP expression, and vice versa (Fig. 1B). Furthermore, analyzing the distribution of the endogenous GIGYF1 and GIGYF2 among native protein complexes by size exclusion chromatography revealed that these two proteins predominantly elute within separate fractions (Fig. 1C). GIGYF2 was primarily eluted in fractions containing complexes of very high molecular masses (>660 kDa) and displayed a similar profile to 4EHP and CNOT1, which represents the core subunit of the Carbon Catabolite Repression—Negative On TATA-less (CCR4-NOT)Carbon Catabolite Repression—Negative On TATA-less (CCR4-NOT) repressor complex, consistent with previous reports that GIGYF2 interacts with subunits of CCR4-NOT complex (*15*). In contrast, GIGYF1 predominantly emerged within lower–molecular mass fractions (~660 kDa), with a different profile to GIGYF2, CNOT1, and 4EHP (Fig. 1C). These differences in elution patterns suggest a divergence in the protein complexes in which GIGYF1 and GIGYF2 participate, their mechanisms of actions, and possibly functional significance.

We previously reported that 4EHP and GIGYF2 repress the translation of the *Ifnb1* mRNA, facilitating replication of RNA viruses (*16*, *28*). Thus, to study the molecular function of GIGYF1, we first evaluated its role in the repression of translation of *Ifnb1*, as a cellular mRNA model, and its impact on viral replication, in comparison with GIGYF2 and 4EHP. We used a human embryonic kidney (HEK) 293 cell model that expresses the human toll-like receptor 3 (TLR3), a receptor recognizing double-stranded viral RNAs. Upon stimulating the cells with polyinosinic-polycytidylic acid [poly(I:C)], a double-stranded RNA mimic and TLR agonist, we measured IFN-β levels in wild-type (WT), 4EHP-KO, GIGYF1-KO, GIGYF2-KO, and GIGYF1/2–double KO (GIGYF1/2-DKO) cells by enzyme-linked immunosorbent assay (ELISA). While both 4EHP-KO and GIGYF2-KO resulted in substantially increased IFN-β production (3.8 ± 0.8–fold and 8.7 ± 1.8–fold increase, respectively) compared to the WT cells, the GIGYF1-KO cells showed a comparatively more profound 33.6 ± 6.2–fold increase in IFN-β production (Fig. 1D). Notably, we observed a slightly augmented increase in IFN-β production in GIGYF1/2-DKO cells, compared with GIGYF1-KO cells. This additional increase further highlights the divergent mechanisms by which GIGYF1 and GIGYF2 repress the translation of their common target mRNAs. Measurement of mRNA expression revealed no notable alternations in *Ifnb1* mRNA levels among any of the indicated genotypes (fig. S2A), suggesting that the observed increased IFN-β production is not due to changes in mRNA expression. A similar exacerbated increase in IFN-β expression in GIGYF1-KO cells, compared with their GIGYF2-KO, 4EHP-KO, and WT counterparts (fig. S2, B and C), and augmented increase in IFN-β production upon concurrent depletion of GIGYF1 and 2 were also observed in A549 human lung epithelial cells (fig. S2, D and E). To determine the impact of GIGYF1 depletion on the translation of *Ifnb1* mRNA, we performed a polysome profiling assay using vehicle or poly(I:C)-treated WT and GIGYF1-KO HEK293 cells. GIGYF1-KO did not affect the global mRNA translation, as evidenced by similar polysome/monosome ratios between the WT and GIGYF1-KO cells in both vehicle- and poly(I:C)-treated conditions (fig. S2, F and G). However, *Ifnb1* mRNA exhibited a marked shift from fractions 4 to 6, which corresponds to poorly translated mRNAs, to fraction 11 that corresponds to association with several ribosomes and therefore highly translated mRNAs in poly(I:C)-treated GIGYF1-KO cells compared to WT cells (Fig. 1E). These findings provide compelling evidence supporting the potent role of GIGYF1 in repressing the translation of *Ifnb1* mRNA.

We proceeded to explore the biological significance of the increased IFN-β production in poly(I:C)-treated GIGYF1-KO cells by measuring the activity of the STAT1 signaling pathway downstream of IFNAR. Western blot (WB) analysis showed enhanced phosphorylation of STAT1 and increased expression of ISG56 in 4EHP-KO and GIGYF2-KO, compared with the WT cells. Notably, GIGYF1-KO cells exhibited a further exacerbated expression of both phospho-STAT1 and ISG56 (Fig. 1F). Next, we investigated whether the GIGYF1-mediated repression of IFN-β production could effectively promote viral replication and dissemination. WT, 4EHP-KO, GIGYGF1-KO, and GIGYF2-KO HEK293 cells were infected with a green fluorescent protein (GFP)–expressing vesicular stomatitis virus (VSV) harboring mutations in the M protein (VSV∆51) and viral replication was measured by viral protein synthesis. Consistent with the elevated levels of IFN-β, phospho-STAT1, and ISG56 expression (Fig. 1F), we observed substantially lower levels of viral protein expression in GIGYF1-KO, compared with the 4EHP-KO and GIGYF2-KO, as well as WT cells (Fig. 1G). Moreover, we used a virus protection assay, a method that evaluates the ability of culture media derived from cells stimulated with poly(I:C) to protect recipient cell populations against virus infection upon transfer of the culture medium (*32*). We treated HEK293 cells of the WT, 4EHP-KO, GIGYF1-KO, and GIGYF2-KO genotypes with poly(I:C) for 6 hours. Following this, we transferred the culture media from the treated cells to recipient HEK293 cells that were left untreated. These recipient cells, referred to as “primed” cells, were subsequently exposed to the VSV∆51-GFP. Viral replication levels were assessed by measuring the GFP signals with fluorescence imaging. Fluorescence imaging (Fig. 1, H and I) indicated substantially lower levels of viral replication in cells primed with media from GIGYF1-KO, compared with those primed with media from 4EHP-KO, GIGYF2-KO, or WT cells. These data underscore that GIGYF1 potently represses IFN-β production, even more efficiently than GIGYF2 and 4EHP, and emphasize that the GIGYF1-mediated repression of IFN-β production is critical for efficient RNA virus replication.

We next examined the role of 4EHP in GIGYF1-mediated robust repression of IFN-β production by generating a mutated isoform (Y39A, Y41A, M46A, and L47A) of GIGYF1 (GIGYF1-Mut) which cannot bind to 4EHP (*12*), as confirmed by coimmunoprecipitation (co-IP) assay (fig. S2H). We complemented the GIGYF1-KO HEK293 cells with either the WT or GIGYF1-Mut constructs (fig. S2I). The 4EHP-binding deficient GIGYF1 very effectively, albeit to a slightly lesser extent than the WT isoform, repressed IFN-β production compared with the GIGYF1-KO cells that expressed GFP as a control (Fig. 1J). This indicates that, in contrast to GIGYF2-mediated repression of *Ifnb1* mRNA that largely relies on 4EHP (*16*), GIGYF1-mediated repression is mainly through a mechanism independent of 4EHP.

### The C-terminal region of GIGYF1 is required for translational repression of target mRNAs

**Fig. 2.**
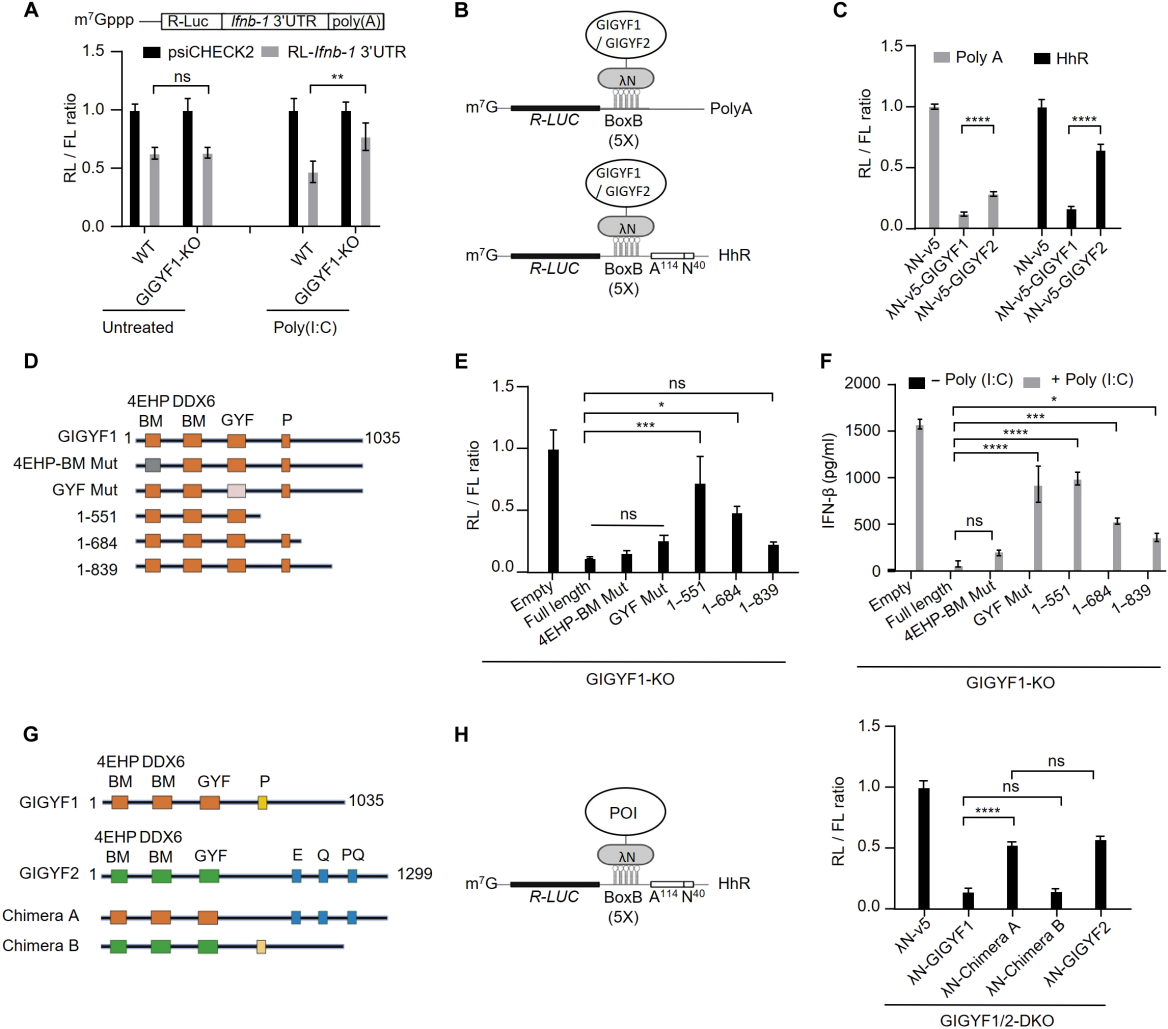
The C-terminal region of GIGYF1 is required for translational repression of target mRNAs. (**A**) Top: Schematic representation of the psiCHECK2-RL-*Ifnb1* 3′UTR reporter. Bottom: WT and GIGYF1-KO HEK293 cells were cotransfected with psiCHECK2-RL-*Ifnb1* 3′UTR reporter or the psiCHECK2-RL reporter without the *Ifnb1* 3′UTR and a firefly luciferase (FL) reporter. Eighteen hours after transfection, cells were mock-treated or stimulated with poly(I:C) (0.05 μg/ml) for 12 hours, followed by measurement of luciferase activities. *Renilla* luciferase (RL) values were normalized against FL, and the ratios were calculated for the psiCHECK2-RL-*Ifnb1* 3′UTR relative to the control psiCHECK2-RL reporter for each condition. (**B**) Schematic representation of the λN:BoxB tether-function system with the deadenylation-permissive RL-5boxB-polyA and deadenylation-resistant RL-5boxB-HhR reporters. (**C**) Analysis of the relative silencing of the RL-5boxB-polyA and RL-5boxB-HhR upon tethering with λN-v5-GIGYF1 or λN-v5-GIGYF2 in HEK293 cells. (**D**) Schematic of the domain structures of WT and mutated GIGYF1 isoforms used in (E) and (F). Images were generated with BioRender.com. (**E**) Tether-function assay in GIGYF1-KO HEK293 cells cotransfected with the indicated plasmid, RL-5BoxB-HhR and FL, followed by dual-luciferase measurement 24 hours after transfection. (**F**) ELISA measurement of IFN-β production in GIGYF1-KO HEK293 cells overexpressing the full-length or indicated mutant GIGYF1 isoforms, following 6 hours of treatment with poly(I:C) (1 μg/ml). (**G**) Schematic domain structures of WT GIGYF1 and GIGYF2 and chimeric constructs derived from the N terminus of GIGYF1 and C terminus of GIGYF2 (Chimera A) or N terminus of GIGYF2 and C terminus of GIGYF1 (Chimera B). Images were generated with BioRender.com. (**H**) Tether-function assay for measurement of repression of the RL-5BoxB-HhR reporter with the indicated constructs in GIGYF1/2-DKO HEK293 cells. Data are presented as mean ± SD (*n* = 3). *P < 0.05, **P < 0.01, ****P < 0.0001; two-way ANOVA [(A) and (C)] or one-way [(E), (F), and (H)] ANOVA with Bonferroni’s post hoc test.

Having established the ability of GIGYF1 to efficiently repress mRNA translation and its important biological role in the regulation of the host cell response to RNA virus infection, we set out to discover the mechanism underlying the efficient and 4EHP-independent translational repression by GIGYF1. 3′ Untranslated regions (3′UTRs) serve as primary binding sites for microRNAs and RBPs, such as TTP, which recruit GIGYF1/2 to the target mRNAs (*33*). We previously showed that the 3′UTR of the *Ifnb1* mRNA mediates the 4EHP-dependent as well as 4EHP-independent translational repression by GIGYF2 (*16*, *28*). Therefore, as an experimental model, we used a reporter system wherein a *Renilla* luciferase (RL) open reading frame was fused to the 3′UTR derived from the human *Ifnb1* mRNA. Compared to the control reporter, the *Ifnb1* 3′UTR construct was repressed by 37 ± 5% in WT cells (Fig. 2A). In the basal untreated condition, GIGYF1 KO did not affect the repression induced by *Ifnb1* 3′UTR (Fig. 2A). However, while poly(I:C) stimulation increased the repression of the *Ifnb1* 3′UTR to 54 ± 9% in the WT cells, this repression was significantly relieved (24 ± 11%, *P* < 0.01; Fig. 2A) in GIGYF1-KO cells. This contrasts with the derepression of the *Ifnb1* 3′UTR construct in untreated conditions in 4EHP-KO and GIGYF2-KO cells (fig. S3A). These data suggest a context-dependent mechanism governing the activation of GIGYF1-mediated repression of IFN-β production that is distinct from the GIGYF2/4EHP-mediated repression.

To gain further mechanistic insights into the robust translational repression by GIGYF1, we used a tether-function assay using the λN:BoxB system (*34*). Briefly, we used an RL reporter containing five BoxB hairpin motifs in its 3′UTR. The reporter was cotransfected into HEK293 cells along with a plasmid encoding a fusion of GIGYF1 or GIGYF2 linked to the λN peptide, which binds to the BoxB hairpins (Fig. 2B, top). Furthermore, to specifically dissect the impact of GIGYF1/2 on mRNA translation, we used a deadenylation-resistant variation of the RL-5BoxB reporter that encodes a self-cleaving Hammerhead ribozyme (HhR) at its 3′ end, generating an internalized Poly(A) stretch of 114 nucleotides to prevent deadenylation and subsequent degradation of the reporter mRNA (Fig. 2B, bottom) (*35*). Despite comparable levels of expression of the λN-GIGYF1 and λN-GIGYF2 proteins (fig. S3B), we observed significantly more repression, particularly of the degradation-resistant HhR reporter, by λN-GIGYF1 compared with λN-GIGYF2 (87.8 and 71.5% repression of the polyA and 83.3 and 35.4% repression of the HhR reporter for GIGYF1 and GIGYF2, respectively; Fig. 2C). We also assessed the kinetics of repression of the RL-5BoxB-polyA and RL-5BoxB-HhR reporters by increasing amounts of λN-GIGYF1 and λN-GIGYF2. With both RL-5BoxB-polyA and RL-5BoxB-HhR reporters, λN-GIGYF1 consistently demonstrated significantly stronger repression compared to λN-GIGYF2 at all tested doses of the plasmids (fig. S3, C and D). These findings further corroborate that, compared with GIGYF2, GIGYF1 exerts greater repression on mRNA translation, irrespective of its susceptibility to deadenylation or the sequence of the target mRNA. Thus, henceforth we exclusively used the HhR system to delineate the mechanisms underlying translational repression by GIGYF1.

To identify the underlying mechanism of robust translational repression of target mRNAs by GIGYF1, we constructed a series of mutants and truncated isoforms of λN-GIGYF1 (Fig. 2D and fig. S3E). Coexpression of these constructs with the RL-5BoxB-HhR reporter in GIGYF1-KO cells revealed that while mutating the 4EHP-binding or GYF motifs had no significant impact on λN-GIGYF1-mediated repression, a progressive truncation of the C-terminal region of GIGYF1 resulted in increasing derepression of the reporter (Fig. 2E). We also tested the impact of the same mutations and truncated isoforms on IFN-β expression in poly(I:C)-treated GIGYF1-KO cells. Similar to the outcomes of the tether-function experiment (Fig. 2E), mutating the 4EHP-binding motif did not significantly affect the repression of IFN-β production by GIGYF1 (Fig. 2F and fig. S3F). The progressive truncation of the C-terminal region of GIGYF1 resulted in an increasing derepression of IFN-β production, compared with the full-length protein (Fig. 2F). However, in contrast to the tether-function assay (Fig. 2E), the GYF motif mutant GIGYF1 isoform failed to significantly repress IFN-β production (Fig. 2F). This discrepancy between the tether-function assay and the repression of IFN-β production can be attributed to the fact that the GYF motif mutant GIGYF1 is incapable of interacting with the RBPs that would otherwise recruit GIGYF1 to the *Ifnb1* mRNA, whereas in the tether-function assay recruitment of GIGYF1 protein to the RL-5BoxB-HhR mRNA is achieved by the λN-tag, rendering the GYF motif dispensable for repression of the reporter mRNA. To further establish the role of the C-terminal region of GIGYF1 in translational repression of target mRNAs, we compared the ability of the full-length and N-terminal or C-terminal truncated isoforms of GIGYF1 (fig. S3H) to repress mRNA translation by tether-function assay. We observed that while deletion of the N-terminal (amino acids 1 to 540) domain, which contains the 4EHP-binding, DDX6-binding, and the GYF motif, only slightly relieved the repression of the target mRNA (88 and 68% repression for the full-length and mutant isoforms, respectively; *P* < 0.0001), deletion of the C-terminal domain (amino acids 418 to 1035) resulted in complete derepression of the target mRNA (fig. S3G). Notably, the C-terminal fragment of GIGYF1 (amino acids 541 to 1035), which lacks the GYF motif and thereby is unable to be recruited to the target mRNAs, failed to rescue the repression of IFN-β expression in GIGYF1-KO cells (fig. S3, I and J). In addition, we created two chimeric constructs by swapping the C-terminal domains of GIGYF1 and GIGYF2 (Fig. 2G and fig. S3K). Whereas the repressive activity of tethered Chimera A containing the N terminus of GIGYF1 and C terminus of GIGYF2 was comparable to the full-length GIGYF2, the repressive activity of tethered Chimera B, consisting of the N terminus of GIGYF2 and C terminus of GIGYF1, was comparable to the full-length GIGYF1 (Fig. 2H).

Together, these data demonstrate that the 4EHP-binding and DDX6-binding motifs of GIGYF1 are largely dispensable for translational repression, the GYF motif is required for recruitment to the target mRNAs through interacting with RBPs but is dispensable for translational repression once GIGYF1 is recruited, and the C-terminal region effects the majority of translational repression, presumably by facilitating interaction with other protein(s).

### GIGYF1 interacts with subunits of eIF3 complex

To further characterize the mechanism underlying GIGYF1-mediated translational repression, we sought to identify its interacting partners using the BioID proximity interactome assay (*36*). We created stable HEK293 cell lines expressing GIGYF1 fused in-frame with an abortive biotin ligase BirA* (R118G) at its N or C terminus (two biological replicates each, four total). We identified 247 unique high-confidence targets for GIGYF1 [false discovery rate (FDR) ≤ 1%], including the known interactors of GIGYF1; TNRC6A, TNRC6B, and TNRC6C, 4EHP (eIF4E2), and ZNF598 (data S1). Gene ontology analysis using the g:Profiler functional enrichment analysis tool revealed protein binding, cytoskeletal protein binding, mRNA binding, and CCR4-NOT complex binding among the top molecular functions enriched in the GIGYF1 proximity interactors identified by our BioID assay (full list available in data S2). Unexpectedly, BioID also unveiled two subunits of the eIF3 complex (eIF3L and eIF3E) among the proximity interactors of GIGYF1. Notably, a recent high-throughput analysis of protein-protein interactions in HEK293T cells also identified subunits of eIF3 as interactors of GIGYF1 (*37*).

**Fig. 3.**
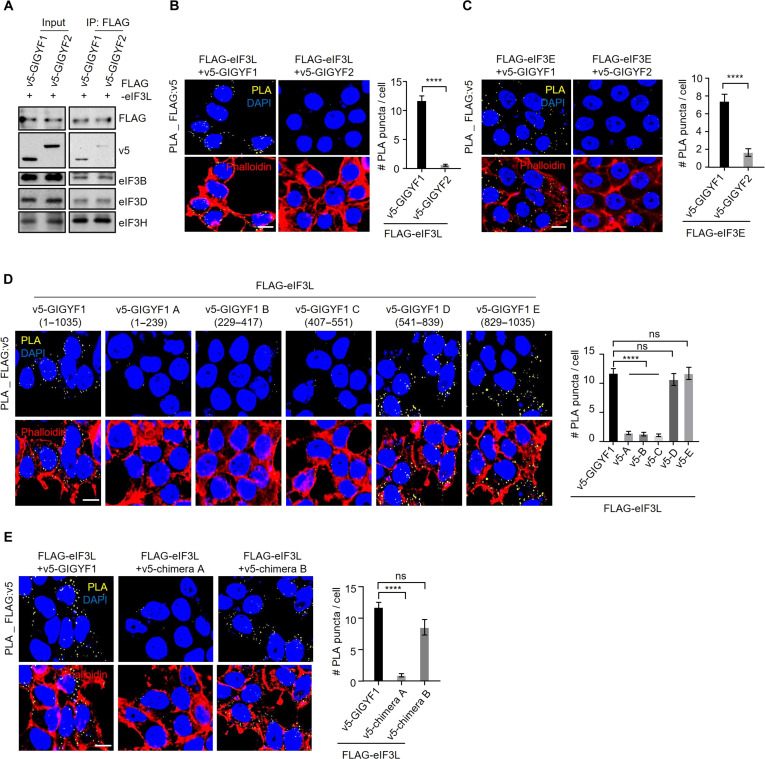
The C-terminal domain of GIGYF1 mediates its interaction with the eIF3 complex. (**A**) Co-IP for detection of interaction between FLAG-eIF3L and indicated proteins in HEK293T cells. Cell lysates were prepared 24 hours after transfection for immunoprecipitation using an anti-FLAG antibody followed by WB with indicated antibodies. (**B** and **C**) Left: PLA for detection of interactions between GIGYF1 and indicated subunits of eIF3 complex. Sites of interactions are visualized as fluorescent punctate in HEK293T cells transfected with vectors expressing v5-GIGYF1 or v5-GIGYF2 together with FLAG-eIF3L (B) or FLAG-eIF3E (C). PLA signals are shown in yellow. The nucleus and actin cytoskeleton were counterstained with 4′,6-diamidino-2-phenylindole (DAPI) and phalloidin (red), respectively. Scale bar, 10 μm. Right: Bar graph represents the number of PLA signals from at least 30 cells, counted in each sample. *n* = 5 independent experiments. (**D**) Left: HEK293T cells were cotransfected with FLAG-eIF3L and the v5-tagged full-length or indicated truncated isoforms of GIGYF1 (see fig. S4K for more details). PLA signals are shown in yellow. The nucleus and actin cytoskeleton were counterstained with DAPI and phalloidin (red), respectively. Scale bar, 10 μm. Right: Bar graph represents the number of PLA signals from at least 20 cells, counted in each sample. *n* = 3 independent experiments. (**E**) Left: PLA for detecting the interactions between eIF3L and the indicated chimeric constructs (described in Fig. 2G). Right: Bar graph represents the number of PLA signals from at least 20 cells, counted in each sample. Scale bar, 10 μm. *n* = 3 independent experiments. Data are presented as mean ± SD (*n* = 3). ****P < 0.0001; unpaired *t* test [(B) and (C)] or one-way ANOVA with Bonferroni’s post hoc test [(D) and (E)].

We verified the interaction between GIGYF1 and eIF3 using co-IP assay, which showed that GIGYF1 coprecipitated with the L, E, D, and G subunits of eIF3 complex with much higher efficiency compared with GIGYF2 (Fig. 3A and fig. S4, A to C). Corroborating the co-IP results, proximity ligation assay (PLA) revealed that cotransfection of v5-GIGYF1 with FLAG-eIF3L, E, D, or G resulted in robust PLA signals, while little or no signals were detected in cells cotransfected with v5-GIGYF2 alongside the examined eIF3 subunits (Fig. 3, B and C, and fig. S4, D to I). Furthermore, size exclusion chromatography analysis of the endogenous proteins revealed that the subunits of the eIF3 complex elute over several fractions, likely a reflection of its involvement with a wide range of complexes engaged in various stages of mRNA translation such as eIF4F, PIC, and reinitiating ribosomes (*38*). However, GIGYF1 mainly coeluted with a fraction of eIF3 proteins characterized by an apparent molecular weight of ~660 kDa (fig. S4J).

To determine the specific region of GIGYF1 responsible for interaction with eIF3 subunits, we engineered five v5-tagged contiguous fragments (designated as GIGYF1 A-E; fig. S4K) and cotransfected them with FLAG-eIF3L in HEK293T cells (fig. S4L). PLA revealed interactions between eIF3L and full-length GIGYF1 as well as fragments D (541 to 839) and E (829 to 1035), both derived from the C-terminal region of GIGYF1 (Fig. 3D). Notably, while cotransfection of FLAG-eIF3L and v5-tagged Chimera A, which encompasses the N-terminal region of GIGYF1 and the C-terminal region of GIGYF2 produced only a weak signal, a strong signal was observed with Chimera B, consisting of the N-terminal of GIGYF2 and C-terminal of GIGYF1 (Fig. 3E and fig. S4M). Similar interaction patterns were also observed for eIF3D with GIGYF1 fragments and Chimeras (fig. S5, A to D). These findings indicate that the region responsible for GIGYF1’s interaction with the eIF3 complex spans its C terminus (residues 541 to 1035), which coincides with the region of the protein that is responsible for the robust repression of the target mRNAs (Fig. 2, E to H).

### GIGYF1 disrupts the interaction between eIF3 complex and eIF4G1

**Fig. 4.**
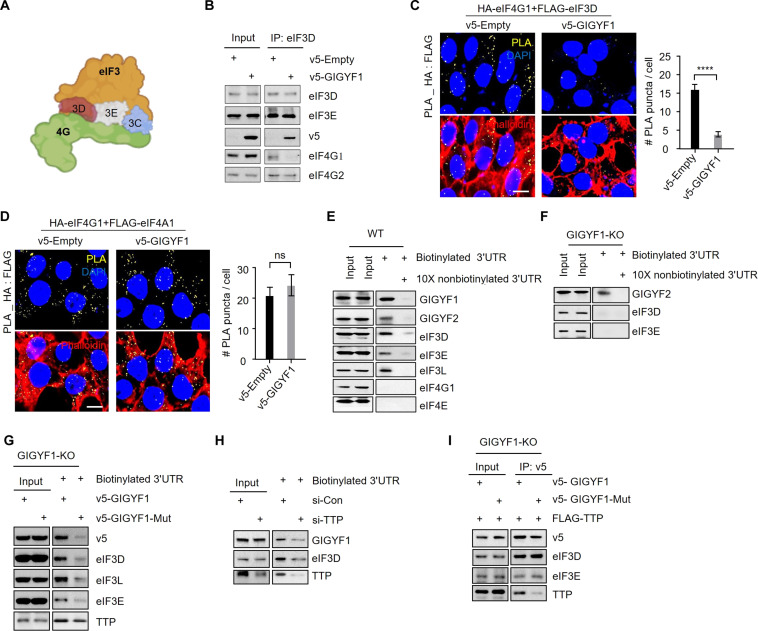
GIGYF1 disrupts the interaction between eIF4G1 and eIF3 subunits. (**A**) Schematic depiction of the subunits of eIF3 at the interface of eIF3-eIF4G1 interaction. Image was generated with BioRender.com. (**B**) Co-IP assay for detection of the impact of ectopically expressed GIGYF1 on interaction between eIF3D and indicated proteins in GIGYF1-KO HEK293 cells. (**C** and **D**) Left: PLA for detection of the impact of ectopic expression of v5-GIGYF1 on eIF4G-eIF3D (C) or eIF4G1-eIF4A1 (D) interactions in GIGYF1-KO HEK293 cells. Twenty-four hours after transfection, cells were fixed and subjected to PLA using HA and FLAG antibodies. Right: Bar graphs represent the number of PLA signals from at least 20 cells, counted in each sample. *n* = 3 independent experiments. Scale bar, 10 μm. Data are presented as mean ± SD (*n* = 3). ****P < 0.0001; unpaired *t* test. (**E** and **F**) Streptavidin-biotin RNA affinity purification assay with biotinylated *Ifnb1* 3′UTR in parental (E) or GIGYF1-KO (F) HEK293 cells. Biotinylated *Ifnb1* 3′UTR was incubated with cell lysates in the presence or absence of 10X nonbiotinylated *Ifnb1* 3′UTR for 16 hours at 4°C. The pulled-down proteins were subjected to Western blotting and probed with the indicated antibodies. (**G**) Streptavidin-biotin RNA affinity purification assay with biotinylated *Ifnb1* 3′UTR and lysates from GIGYF1-KO cells expressing v5-GIGYF1 or GYF motif mutant v5-GIGYF1. (**H**) Streptavidin-biotin RNA affinity purification assay with biotinylated *Ifnb1* 3′UTR in TTP-depleted cells. Biotinylated *Ifnb1* 3′UTR was incubated with lysates derived from HEK293 cells treated with control siRNA (si-Con) or si-TTP, followed by Western blotting and probing with the indicated antibodies. (**I**) Co-IP assay for detection of the impact of mutating the GYF motif on the interaction between GIGYF1, eIF3D, eIF3E, and TTP in GIGYF1-KO HEK293 cells.

We next set out to investigate whether the interactions between GIGYF1 and eIF3 play a role in the translational repression of target mRNAs by GIGYF1. Our BioID, PLA, and co-IP data consistently demonstrated that GIGYF1 interacts with eIF3 through its C-terminal region (Fig. 3), which is also responsible for orchestrating potent translational repression of mRNAs upon recruitment of GIGYF1 (Fig. 2, E to H). Binding of eIF3 to eIF4G is instrumental in the recruitment of PIC to the mRNA 5′ cap. Previous structural analyses of the interactions between the eIF3 complex and eIF4G have revealed that the E, D, and C subunits of eIF3 constitute the interface through which it binds to eIF4G [Fig. 4A; (*39*)]. Therefore, we postulated that GIGYF1 represses the translation of its target mRNAs by interacting with eIF3 at the interface where it binds to eIF4G, effectively disrupting the critical eIF3-eIF4G interaction. This disruption could abrogate the binding of PIC to eIF4F on the mRNA cap, subsequently hindering mRNA translation initiation. To test this hypothesis, we first examined the impact of GIGYF1 on interactions between subunits of eIF3 and eIF4G by co-IP. Restoring GIGYF1 expression in GIGYF1-KO HEK293 cells reduced the coprecipitation of eIF4G1, but not eIF3E, by eIF3D (Fig. 4B and fig. S6A). GIGYF1 overexpression, however, did not affect eIF3D-mediated pulldown of eIF4G2 (DAP5), a member of the eIF4G proteins family, which binds to eIF4A and eIF3, but not eIF4E, and thereby drives cap-independent translation (*40*). This may be due to the absence of GIGYF1 recruitment to the mRNAs that use DAP5 for cap-independent translation.

GIGYF1 overexpression did not affect eIF3D-mediated pulldown of eIF2α, a key subunit of PIC (fig. S6B), consistent with the specificity of GIGYF1-mediated interruption of eIF3 interaction with eIF4G1. GIGYF1 overexpression, however, enhanced coprecipitation of TTP with eIF3D (fig. S6B). TTP is an RBP that is known to regulate the translation as well as stability of its target mRNAs through interactions with the GYF motifs of GIGYF1/2 (*41*). This indicates that RBPs that recruit GIGYF1 to their target mRNAs could form complexes that also involve eIF3 via GIGYF1. Moreover, we observed that increasing amounts of GIGYF1 expression in GIGYF1-KO cells resulted in a reduced coprecipitation of eIF4G1 but not eIF3E by eIF3D (fig. S6C). Similar results were obtained with co-IP using eIF3E, which showed a reduced association with eIF4G1 but not eIF3D upon expression of GIGYF1 in GIGYF1-KO cells (fig. S6D). Overexpressing GIGYF1 also led to a diminished PLA signal between HA-eIF4G1 and FLAG-eIF3D, but not between HA-eIF4G1 and FLAG-eIF4A1, components of the eIF4F complex (Fig. 4, C and D, and fig. S6, E and F). These findings indicate that GIGYF1 ectopically expressed in GIGYF1-KO HEK293 cells selectively disrupts the association between the eIF3 complex and eIF4G1 while leaving intact the interactions between the different subunits of eIF3, PIC and eIF3, and the subunits of the eIF4F complex.

We next queried whether the interaction between GIGYF1 and eIF3 occurs within the context of the mRNAs that recruit GIGYF1 by using a streptavidin-based pulldown assay with biotinylated *Ifnb1* 3′UTR (without cap or open reading frame). We observed that besides GIGYF1 and GIGYF2, the biotinylated *Ifnb1* 3′UTR also precipitated eIF3L, E, and D but not eIF4G1 or eIF4E (Fig. 4E). Pulldown of these factors with biotinylated *Ifnb1* 3′UTR was markedly reduced upon the introduction of a competitor nonbiotinylated *Ifnb1* 3′UTR (Fig. 4E), indicating the specificity of the biotinylated *Ifnb1* 3′UTR-mediated pulldown. To ensure that the absence of eIF4E and eIF4G1 pulldown with the biotinylated *Ifnb1* 3′UTR was not due to a technical error, we used a capped version of the same biotinylated *Ifnb1* 3′UTR. We observed similar levels of pulldown of both eIF4E and eIF4G by the capped biotinylated *Ifnb1* 3′UTR in WT and GIGYF1-KO cells (fig. S6, G and H). Notably, we did not observe any visible precipitation of eIF3D or eIF3E in GIGYF1-KO cells by biotinylated *Ifnb1* 3′UTR, despite the evident pulldown of GIGYF2 by the biotinylated *Ifnb1* 3′UTR (Fig. 4F). This substantiates that association of eIF3 subunits with *Ifnb1* 3′UTR is GIGYF1 dependent.

We next examined whether the GYF motif of GIGYF1, which facilitates the GYF:PRS-mediated interaction with RBPs such as TTP, is necessary for the interaction between eIF3 and target mRNAs. We observed that compared with the WT GIGYF1, mutation of the GYF motif led to a reduction in the recruitment of GIGYF1 and subunits of eIF3 complex (L, E, and D) to *Ifnb1* 3′UTR (Fig. 4G and fig. S6I). Mutating the GYF motif of GIGYF1 had no impact on the association of TTP (Fig. 4G and fig. S6I), which is known to directly bind to the A/U-rich elements in the 3′UTR of the *Ifnb1* mRNA (*42*). Conversely, small interfering RNA (siRNA)–mediated knockdown of TTP resulted in substantially reduced precipitation of GIGYF1 and eIF3D with biotinylated *Ifnb1* 3′UTR, which confirms TTP as an RBP that can recruit GIGYF1 to the *Ifnb1* mRNA 3′UTR (Fig. 4H). Notably, mutating the GYF motif diminished the interaction between GIGYF1 and TTP, but it did not detectably abrogate the interaction between GIGYF1 and eIF3E or eIF3D (Fig. 4I), indicating that the GYF motif is crucial for recruitment to the target mRNA but not for interaction with eIF3. This aligns with our previous finding that eIF3 interacts with the C terminus of GIGYF1 (Fig. 3, D and E) and that the C-terminal region, rather than the GYF domain, is responsible for the GIGYF1-mediated repression (Fig. 2, E to H). Unlike the full-length protein, neither the N-terminal fragment (amino acids 1 to 239), nor the C-terminal fragments (amino acids 541 to 839, amino acids 829 to 1035, or amino acids 541 to 1035) of GIGYF1 were able to induce a significant reduction in the eIF3D:eIF4G1 interaction upon overexpression in the GIGYF1-KO cells (fig. S7, A to D). These data further suggest that the disruption of the eIF4G1/eIF3 interaction on the mRNA cap by GIGYF1 requires the initial recruitment of the GIGYF1 protein to mRNA, presumably via GYF motif.

### GIGYF1 disrupts the eIF3/eIF4G interaction in a context-dependent manner

**Fig. 5.**
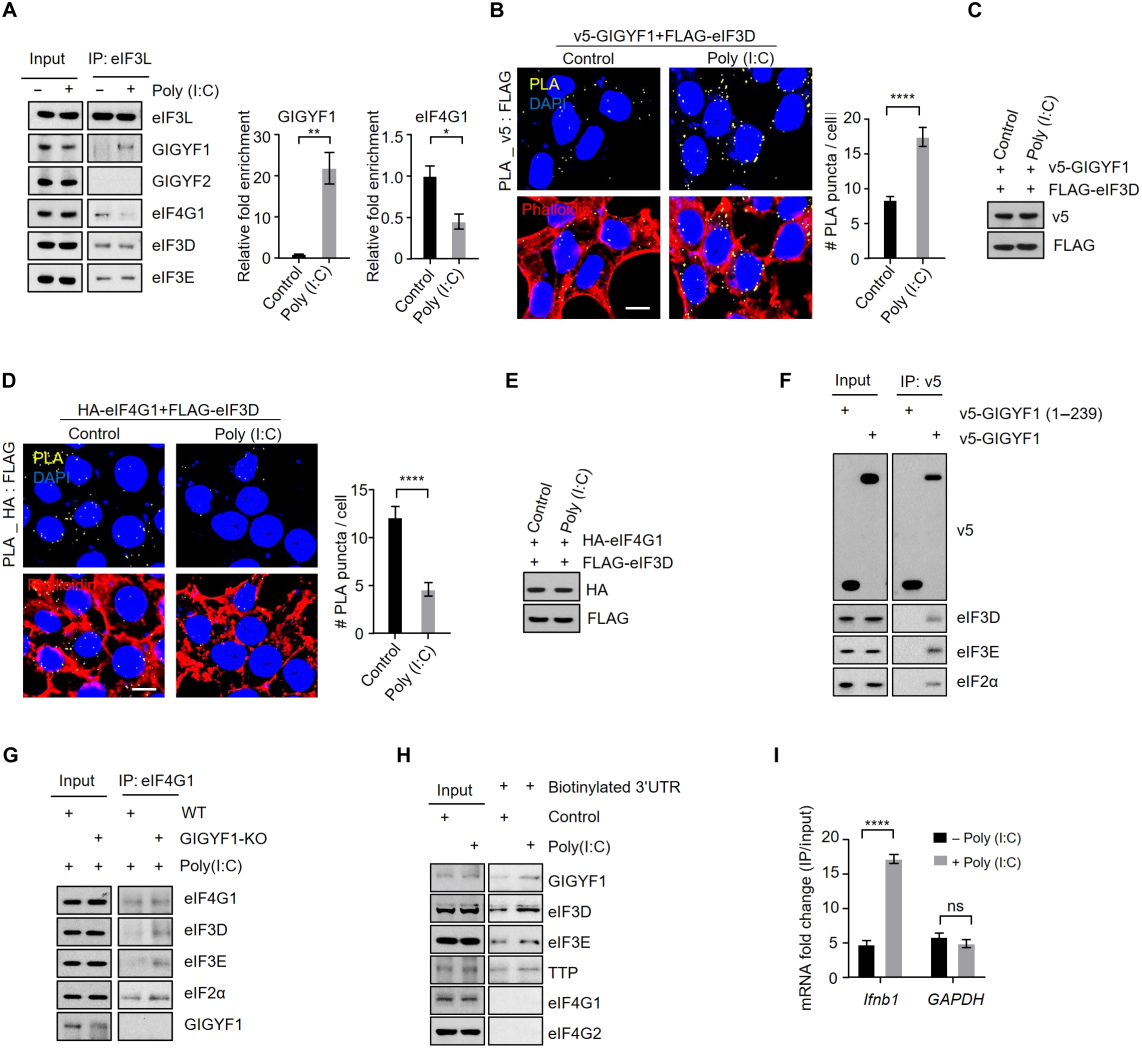
Poly(I:C) stimulation induces GIGYF1-mediated disruption of the eIF3/eIF4G1 interaction. (**A**) Left: Co-IP for detection of interaction between endogenous eIF3L and GIGYF1 or eIF4G1 upon poly(I:C) stimulation. Right: Quantification of the indicated coprecipitated proteins, normalized to eIF3L. *n* = 3 independent experiments. (**B**) Left: PLA for detection of interactions between v5-GIGYF1 and FLAG-eIF3D upon poly(I:C) stimulation. PLA signals are shown in yellow; the nucleus and actin cytoskeleton were counterstained with DAPI and phalloidin (red), respectively. Scale bar, 10 μm. Right: Bar graph represents the number of PLA signals from at least 20 cells, counted in each sample. *n* = 3 independent experiments. (**C**) WB analysis of cell lysates from (B). (**D**) Left: PLA for detection of interactions between HA-eIF4G1 and FLAG-eIF3D upon poly(I:C) stimulation. Right: Bar graph represents the number of PLA signals from at least 20 cells, counted in each sample. Scale bar, 10 μm. *n* = 3 independent experiments. (**E**) WB analysis of cell lysates from (D). (**F**) Co-IP assay for detection of the interaction between GIGYF1, eIF3D, and eIF2α in GIGYF1-KO HEK293 cells transfected with v5-tagged full-length or N-terminal fragment (amino acids 1 to 239) of GIGYF1. (**G**) Co-IP assay for detection of the interaction between endogenous eIF4G1, eIF3, and eIF2α proteins in WT and GIGYF1-KO cells upon poly(I:C) stimulation. (**H**) Biotinylated *Ifnb1* 3′UTR was incubated with lysates from vehicle or poly(I:C)-treated HEK293 cells. The pulled-down proteins were subjected to WB and probed with the indicated antibodies. (**I**) RNA-IP and qRT-PCR analysis of the association between v5-GIGYF1 and *Ifnb1* mRNA in vehicle or poly(I:C)-treated HEK293 cells. Data are presented as mean ± SD (*n* = 3). *P < 0.05, **P < 0.01, ****P < 0.0001; unpaired *t* test [(A), (B), and (D)] or two-way ANOVA with Bonferroni’s post hoc in (I).

Subsequently, we investigated whether the poly(I:C)-stimulated translational repression by GIGYF1 (Fig. 2A) affects the interactions between eIF3 and GIGYF1 or eIF4G1. Co-IP assay with endogenous eIF3L protein demonstrated that while poly(I:C) treatment did not have an effect on interactions between eIF3E and eIF3D with eIF3L, it substantially increased coprecipitation of GIGYF1, while reducing coprecipitation of eIF4G1 (Fig. 5A). In contrast, poly(I:C) treatment did not have a tangible effect on pulldown of eIF4G1 by eIF3L in GIGYF1-KO cells (fig. S8A). We also observed a significantly augmented PLA signal between v5-GIGYF1 and FLAG-eIF3D (Fig. 5, B and C) or FLAG-eIF3G (fig. S8, B and C) upon poly(I:C) stimulation. In contrast, poly(I:C) stimulation significantly decreased the PLA signal between FLAG-eIF3D and HA-eIF4G1 (Fig. 5, D and E).

We next tested whether the interaction between GIGYF1 and eIF3 occurs in the context of the PIC/eIF4F complex that typically forms on the mRNA 5′ cap as part of the cap-dependent translation initiation process. Co-IP assay revealed that besides eIF3D, the full-length GIGYF1 is able to also pulldown eIF2α, a main component of PIC (Fig. 5F). Furthermore, co-IP with endogenous proteins in poly(I:C)-treated WT and GIGYF1-KO cells showed that GIGYF1 depletion substantially increased pulldown of eIF3D and eIF2α proteins with eIF4G1 (Fig. 5G). These data suggest that GIGYF1 is able to interact with eIF3 in the context of the PIC/eIF4F complex and abrogate this complex by disrupting the crucial eIF3/eIF4G1 interaction. Our luciferase reporter assay using the *Ifnb1* mRNA 3′UTR had shown that GIGYF1-KO only affects the repression mediated by this 3′UTR in the context of poly(I:C) stimulation (Fig. 2A). We hypothesized that this context-dependent effect may be due to increased recruitment of GIGYF1 to this mRNA upon poly(I:C) stimulation. We tested this hypothesis using streptavidin-based pulldown of biotinylated *Ifnb1* 3′UTR in control and poly(I:C)-treated cells and observed a substantially increased pulldown of GIGYF1, eIF3E, and eIF3D, but not TTP, in poly(I:C)-treated cells (Fig. 5H). We did not detect pulldown of eIF4G1 or eIF4G2 in either condition (Fig. 5H). Consistently, RNA immunoprecipitation (RNA-IP) in control and poly(I:C)-treated cells revealed significantly more (threefold; *P* < 0.001) pulldown of the endogenous *Ifnb1* mRNA with v5-GIGYF1 upon poly(I:C) stimulation (Fig. 5I and fig. S8D).

## DISCUSSION

**Fig. 6.**
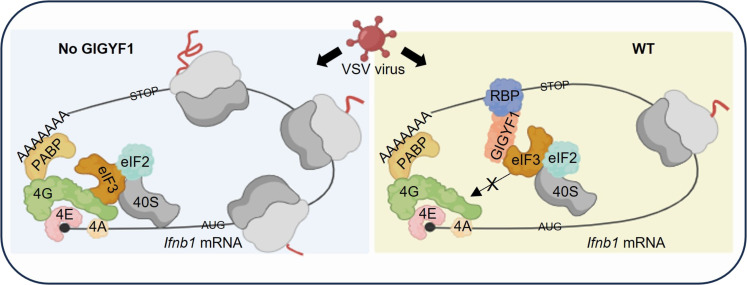
The mechanism of GIGYF1-mediated repression of the cap-dependent mRNA translation initiation. Proposed model for mechanism of translational repression of the *Ifnb1* mRNA by GIGYF1. Left: Cap-dependent translation initiation involves the recognition of the m^7^G cap by the eIF4F complex, comprising eIF4E, eIF4A, and eIF4G1. The eIF3 complex serves as a bridge between eIF4G1 and the PIC. Upon recruitment by eIF3, the PIC scans the 5′UTR until it reaches the translation start codon. Subsequently, it recruits the 60*S* ribosomal subunit, leading to the assembly of the 80*S* ribosome and the beginning of the elongation phase. Right: Upon recruitment to an mRNA by RBPs, typically through GYF:PRS motifs, the C-terminal region of GIGYF1 binds to the subunits of eIF3 at the interface of the eIF3:eIF4G interaction. This binding effectively disrupts the crucial eIF3/eIF4G interaction, thereby repressing mRNA translation initiation. Images were generated with BioRender.com.

Our findings collectively support a model in which upon recruitment to a target mRNA via a RBP, GIGYF1 inhibits mRNA translation initiation by selective disruption of the interaction between eIF3 and eIF4G1 (Fig. 6). In the past few decades, several mechanisms of regulation of general cap-dependent mRNA translation initiation have been described (*43*). These include blocking the eIF4G and eIF4E interaction by the small eIF4E-binding proteins (4E-BPs) and inhibiting the formation of the PIC complex through phosphorylation of eIF2α. In addition, previous studies also revealed transcript-specific mechanisms of regulation of translation initiation. For instance, repression of terminal oligopyrimidine mRNAs and microRNA target mRNAs occurs through the displacement of eIF4E from the cap by LARP1 and 4EHP, respectively (*44*–*46*). Furthermore, RBPs such as IRP-1 (*47*) and L13a (*48*) have been shown to hinder transcript-specific recruitment of PIC to eIF4G, thereby blocking mRNA translation. This study represents a unique mechanism of transcript-specific regulation of translation initiation through the binding of a regulatory protein—i.e., GIGYF1—to eIF3, and specific disruption of its interaction with eIF4G1. Our data indicate that GIGYF1 can disrupt the eIF4G1/eIF3 interaction in the context of the eIF4F/PIC complex (Fig. 5, F to H). However, a limitation of this model is the possibility of additional eIF3/PIC recruitment to the vacant eIF4F to initiate translation on the GIGYF1 target mRNA. It is plausible that GIGYF1-mediated disruption of eIF3/eIF4G1 creates a localized imbalance in the ratio of available PIC to cap-bound eIF4F and therefore represses translation initiation. Another plausible mechanism involves phase-separated membraneless RNA granules. Accordingly, the RBP-GIGYF1 mediated disruption of the eIF4G1/eIF3 interaction could occur within or promote the formation of RNA granules, which limit the availability of free PICs. GIGYF1, TTP, as well as several other GIGYF1-interacting proteins identified in our proximity interactome assay (data S1), have been found in cytoplasmic RNA granules associated with translation repression (*49*).

This translational repression mechanism is transcript-specific and is likely used by RBPs such as TTP, TNRC6s, and ZNF598 that bind to the GYF domain of GIGYF1 through their PRSs. We demonstrated that TTP is a likely candidate for recruitment of GIGYF1 to *Ifnb1* mRNA 3′UTR. The recruitment of GIGYF1 via RBPs to a target mRNA and the subsequent translational repression through abrogation of eIF3/eIF4G1 interaction could also be influenced by other factors. For instance, poly(I:C) stimulation significantly enhances recruitment of GIGYF1 to *Ifnb1* mRNA, which, along with the increased GIGYF1:eIF3L and reduced eIF3:eIF4G1 interactions in poly(I:C)-stimulated cells, provides a plausible mechanism for the transcript-specific regulation of *Ifnb1* mRNA. This is further highlighted by the observation that GIGYF1 abrogates the interaction between eIF3 and eIF4G1, but not eIF4G2 (DAP5), despite the substantial homology of DAP5 to eIF4G1 in the central segment of eIF4G1, which corresponds to the eIF4A and eIF3 binding region (*50*). The distinct impacts of GIGYF1 on interactions of eIF3 with eIF4G1 and eIF4G2 is likely due to the recruitment of GIGYF1 by RBPs to mRNAs that use eIF4G1 mediated cap-dependent initiation, rather than the eIF4G2 mediated cap-independent initiation. Because of the broad spectrum of potential targets arising from the interactions of GIGYF1 with miRISC, ZNF598, TTP, and other RBPs, as evidenced by our BioID data (data S1), it is expected that the specific set of cellular mRNAs regulated through the GIGYF1-eIF3 interaction mechanism is diverse and context dependent. Future studies using high-throughput assays such as Ribo-Seq could unveil the comprehensive repertoire of the translatome regulated by the intricate interplay among RBPs/GIGYF1/eIF3 complexes under different conditions that induce the expression or activity of these RBPs and their target mRNAs.

Our previous study (*44*) suggested that, similar to GIGYF1, 4EHP may also coexist within the same complex as eIF3E and eIF3L. This raises an intriguing possibility that GIGYF1 may use a bifurcated mechanism to repress cap-dependent translation of target mRNAs: (i) direct disruption of the eIF3/eIF4G1 interaction and (ii) utilization of 4EHP to displace eIF4E from the cap structure. Notably, GIGYF1 and GIGYF2 have very similar GYF motifs, which enables them to interact with similar types of RBPs such as TTP, ZNF598, and the miRISC complex. Considering that many mRNAs have multiple binding sites for microRNAs and RBPs that could recruit GIGYF1 and GIGYF2, it is plausible that an mRNA could recruit and be simultaneously regulated by a combination of these mechanisms (i.e. GIGYF1-eIF3, GIGYF1-4EHP, and GIGYF2-4EHP). The implications and relative contribution of each mode of translational repression exerted by these proteins under different conditions require further investigation in future studies.

The complex between GIGYF2 and 4EHP serves as a key player in the translational repression triggered by microRNAs and RBPs (*12*, *15*, *17*, *41*, *51*). While the mechanism by which GIGYF1 regulates mRNA translation has been poorly characterized, compelling evidence underscores that GIGYF2 can also exert substantial translational repression through mechanism(s) that operate independently of 4EHP (*15*, *16*). Our experimental data has affirmed that, similar to GIGYF2, GIGYF1 also interacts with 4EHP. However, in contrast to GIGYF2, which relies on its interaction with 4EHP for approximately 50% of the translational repression of target mRNAs (*16*), the interaction with 4EHP only contributes to a small fraction of GIGYF1-mediated translational repression (Figs. 1J and 2, E and F). No empirically validated structures are available for either GIGYF1 or GIGYF2 proteins. Nevertheless, despite sharing a high degree (55%) of similarity of amino acid sequences, analysis of their predicted domain structures has revealed conspicuous differences in their C-terminal regions (fig. S1). This may explain their differing abilities to interact with eIF3 subunits and use those interactions for repression of target mRNAs. We noted that both the amino acid 541 to 839 and amino acid 829 to 1035 fragments of GIGYF1 are independently capable of interacting with the L and D subunits of eIF3 (Fig. 3D and fig. S5A). This suggests that GIGYF1 may have multiple interaction sites with either the same or different subunits of eIF3. Consequently, further investigations are warranted to determine the structural characteristics of the C-terminal domains of GIGYF1 and GIGYF2 and elucidate the mechanism underlying their differential interactions with eIF3. These studies may also have implications in identifying potential sites for developing small-molecule inhibitors or competitive peptides that disrupt the GIGYF1-eIF3 interaction. Such interventions could be valuable in modulating the immune system, particularly in scenarios where an overactive immune response leads to tissue damage.

We demonstrate that, through binding to eIF3, GIGYF1 represses the translation of *Ifnb1* mRNA, which encodes the crucial immunoregulatory cytokine IFN-β. This regulatory action effectively restrains the extent of activation of the host cell’s innate antiviral immune response to RNA virus infection. Congruently, depletion of GIGYF1 conferred resistance to VSV virus replication, primarily due to the enhanced IFN-β production, indicating its significance in facilitating viral replication. Thus, both GIGYF1 and GIGYF2 play a role in the suppression of the innate immune system upon RNA virus infections, although through divergent mechanisms. This function of the GIGYF family appears to be evolutionarily conserved. The depletion of SMY2-type ILE-GYF domain-containing protein 1 (EXA1), the plant homolog of GIGYF1, has also been identified as a susceptibility gene for viral infections (*52*–*54*).

Viruses use various strategies to suppress the host’s cap-dependent mRNA translation and redirect the cellular ribosomes toward viral mRNAs (*55*). Among the translation factors frequently exploited by viruses to inhibit host protein synthesis are eIF4G and eIF3. These encompass the degradation or cleavage of eIF4G1 (*56*, *57*), disruption of interactions between eIF4G1 and the poly(A) binding protein (*58*), or sequestration of eIF4F subunits in cytoplasmic “viral factories” (*59*). Several viral factors hinder host cap-dependent initiation by encoding proteins that bind to subunits of the eIF3 complex, thus interrupting its interactions with PIC (*60*–*63*). An alternative mechanism has been reported for Sendai virus, where it boosts the production of the host IFN-stimulated genes ISG56 and ISGP54, which in turn inhibit translation initiation through binding to eIF3C and eIF3E, antagonizing eIF2-GTP-Met-tRNAi loading, and preventing the PIC recruitment to eIF3 (*64*–*66*). We previously reported that the SARS-CoV-2–derived NSP2 protein represses translation of *Ifnb1* (*16*) and other mRNAs (*67*) through co-opting the GIGYF2/4EHP complex. However, NSP2 does not interact with GIGYF1 (*16*, *30*, *31*). Therefore, it remains to be understood whether viruses directly use GIGYF1 to disrupt the eIF4G1-eIF3 interaction and regulate host cell mRNA translation, either in a general or transcript-specific manner. Alternatively, viruses may co-opt this mechanism by enhancing the expression or activity of RBPs that recruit GIGYF1 for such regulatory purposes. Notably, enhanced expression of TTP, which enables recruitment of GIGYF1 to *Ifnb1* mRNA, has been observed upon infection with several types of viruses (*68*–*71*).

Disrupting the RBP/GIGYF1/eIF3 complex enhances the host antiviral response, as evidenced by increased IFN-β production, and reduced viral replication observed in GIGYF1-KO cells. Recent research has unveiled that GIGYF1 can undergo phosphorylation at multiple residues, including S638, located near the GYF motif (amino acids 502 to 504), mediated by p38 kinase and c-Jun N-terminal kinase (JNK). S638 phosphorylation inhibits the GYF:PRS-mediated interactions between GIGYF1 and RBPs such as TTP and ZNF598 (*72*). Both p38 kinase and JNK have been implicated in the host antiviral response (*73*–*75*). Conversely, excessive activation of p38 can lead to cytokine storm and tissue damage (*73*, *76*) and pharmacological inhibition of p38 has been shown to suppress the expression of proinflammatory cytokines, including IFN-β, in cells infected with SARS-CoV-2 (*73*). Thus, it is plausible that under resting conditions, the RBP/GIGYF1/eIF3 axis maintains mRNAs that encode the proinflammatory cytokines such as IFN-β in a translationally repressed state. Upon stimulation of the innate immune system, the interactions between GIGYF1 and eIF3 increase, thereby disrupting the eIF4G1-eIF3 interactions (Fig. 4, H and I). This is potentially due to the enhanced expression of mRNAs (e.g., *Ifnb1*) that harbor binding sites for RBPs (e.g., TTP) that recruit GIGYF1 and thereby elevate the frequency of GIGYF1-eIF3 interactions. An alternative possibility includes posttranslational modifications or interactions with additional factors that further stimulate GIGYF1-eIF3 binding. Conversely, S638 phosphorylation of GIGYF1 and the subsequent disruption of its interaction with RBPs (e.g., TTP) provide a mechanism for rapid derepression of translation of mRNAs encoding antiviral proteins, such as IFN-β and mounting the antiviral immune response by the host cells. However, the potential role of the RBP/GIGYF1/eIF3 mechanism of translational control and the p38 and JNK-mediated S638 phosphorylation of GIGYF1 in maintaining the delicate balance between promoting the antiviral response and preventing an unrestrained proinflammatory reaction during viral infections necessitates further investigation in future studies.

In summary, our study reveals that GIGYF1 plays a crucial role in repressing cellular mRNA translation through a distinctive, transcript-specific mechanism. This mechanism entails GIGYF1’s recruitment to the mRNA through interactions with RBPs, followed by its binding to eIF3 subunits and displacing eIF4G1 from the eIF3 complex. Our findings also highlight the biological significance of this mechanism, as it effectively dampens the antiviral immune response and facilitates RNA virus infections. These results indicate the potential utility of this mechanism in devising effective methods to manipulate important biological processes such as antiviral immune response and autoimmune disorders.

## MATERIALS AND METHODS

### Cell line, cell culture, and transient transfection

HEK293T (Thermo Fisher Scientific) cells were maintained in Dulbecco’s modified Eagle’s medium (DMEM) (Wisent Inc., 319-005-CL) supplemented with 10% fetal bovine serum (FBS) (R&D Systems, S12450) and 1% penicillin/streptomycin (P/S) (Wisent Technologies). A549 (American Type Culture Collection), were maintained in RPMI 1640 (Wisent Inc., 350-000-CL), also supplemented with 10% FBS and 1% P/S. WT, 4EHP-KO, GIGYF1-KO, GIGYF2-KO, and GIGYF1/2-DKO HEK293 cells were maintained in DMEM supplemented with 10% FBS, 1% P/S, Zeocin (100 μg/ml; Thermo Fisher Scientific, R25001) and blasticidin (15 μg/ml; Thermo Fisher Scientific, R210-01). All cells were cultured in a humidified atmosphere of 5% CO_2_ at 37°C.

### Antibodies, siRNAs, and plasmids

The following antibodies were used: rabbit anti-GIGYF1(Bethyl, A304-132A), rabbit anti-GIGYF2 (Bethyl, A303-732A-M), rabbit anti-eIF4E2 (Genetex, GTX82524), mouse anti-β-actin (Sigma-Aldrich, A5441), rabbit anti-TLR3 (Cell Signaling Technology, 6961), rabbit anti–phospho-STAT1 (Tyr^701^; Cell Signaling Technology, 7649), rabbit anti-STAT1 (Cell Signaling Technology, 14994), anti-ISG56, rabbit anti–VSV glycoprotein (VSV-G) (Abcam, ab3556), mouse anti-v5 (Abcam, ab27671), mouse anti–hemagglutinin (HA) (Biolegend, 901515), rabbit anti-HA (Sigma-Aldrich, H6908), mouse anti-FLAG (Abcam, ab49763), rabbit anti-FLAG (Sigma-Aldrich, F7425), rabbit anti-eIF3D (Abcam, ab155419, ab264228), rabbit anti-eIF4G1 (Cell Signaling Technology, 2858), rabbit anti-eIF3E (Abcam, ab36766), rabbit anti-eIF3L (Bethyl Laboratories, A304-754A), mouse anti-eIF4E (BD Biosciences, 610270), rabbit anti-TTP (Cell Signaling Technology, 71632), rabbit anti-eIF4G2 (Cell Signaling Technology, 3468), rabbit anti-eIF3H (Cell Signaling Technology, 3413), rabbit anti-eIF3B (Bethyl, A301-761A), and rabbit anti-CNOT1 (Proteintech, 14276-1-AP).

The following siRNAs were used in this study: ON-TARGETplus Nontargeting Control Pool (Dharmacon, D-001810-10-05), GIGYF2 siRNA SMARTpool (Dharmacon, L-013918-01-0005), and ZFP36/TTP siRNA SMARTpool (Dharmacon, L-010789-01-0005). The psiCHECK-2 vector (Promega, C8021) and the psiCHECK-RL-*Ifnb1* 3′UTR vector were described before (*28*). Plasmids encoding λN-v5-GIGYF1, λN-v5-GIGYF2, λN-v5-GIGYF1 truncations, Chimera A, and Chimera B were generated by cloning into pCI-neo-λN-v5 vector using Xho I and Not I restriction sites. The 4EHP-binding mutant GIGYF1 (GIGYF1 Mut; Y39A, Y41A, M46A, and L47A), GYF motif mutant GIGYF1 (GIGYF1 GYF Mut; G502A, Y503A, and F504A) plasmids were created using the QuikChange II Site-Directed mutagenesis kit (Agilent Technologies, 200523). The chimeric constructs were synthesized by GenScript (Piscataway, NJ, USA), and subcloned into the pCI-neo-λN-v5 vector using Xho I and Not I restriction sites. The sequence of the primers used are listed in table S1.

### Generation of KO cell lines by CRISPR-Cas9

The 4EHP-KO and GIGYF2-KO A549 cells, 4EHP-KO, GIGYF2-KO, and GIGYF1/2-DKO HEK293 cells were described previously (*16*, *28*). The GIGYF1-KO A549 cells were generated using Lenti-Cas9-Blast (Addgene, plasmid 52962) and pLenti-CRISPRv2 (Addgene, plasmid 52961). Sequences of the small guide RNAs are listed in table S1.

### Virus production

Lentivirus pseudovirions were produced by transfecting HEK293T cells using Lipofectamine 2000 and 10 μg of Lentiviral plasmid, 6 μg of psPAX2 (Addgene, plasmid 12260) and 4 μg of pMD2.G (Addgene, plasmid 12259). Forty-eight hours after transfection, supernatants were collected from which pseudovirions were purified by ultracentrifugation (32,000 rpm) for 2 hours. The pelleted virus was resuspended into the DMEM. Virus titer was adjusted to 5 multiplicities of infection (MOIs) before storage at −80°C. Single-cycle replication-competent pseudovirions were generated by cotransfecting pVSV-eGFP-DG (Addgene, 31842) with VSV-G–expressing plasmids into HEK293T cells. Forty-eight hours after transfection, pseudovirus was purified from the cell culture supernatant by ultracentrifugation (35,000 rpm for 2 hours at 4°C). The pelleted virions were suspended for future experiments.

### Virus protection assay

VSV protection assay was performed as previously described (*32*). WT, 4EHP-KO, GIGYF1-KO, and GIGYF2-KO HEK293 cells were either mock treated or transfected with poly(I:C) (1 μg/ml; Sigma-Aldrich, P1530) using Lipofectamine 2000. Supernatant was collected after 6 hours and added to the recipient HEK293 cells. After overnight incubation, primed cells were infected with VSVΔ51-GFP at 0.01 MOI for 12 hours. Virus replication was visualized using WB and fluorescence microscopy.

### Poly(I:C) treatment and ELISA

HEK293 and A549 cells were seeded at 80 to 90% confluency. Cells were treated for 6 hours with the indicated concentration of high–molecular weight poly(I:C) (InvivoGen, tlrl-pic) using Lipofectamine 2000. In experiments involving TLR3-expressing cells, poly(I:C) treatment (Sigma-Aldrich, P1530) was administered instead. IFN-β amounts in the culture supernatant were measured by the human IFN-β ELISA kit (R&D Systems, DIFNB0) according to the manufacturer’s protocol.

### Dual-luciferase reporter assays

WT, 4EHP-KO, GIGYF1-KO, and GIGYF2-KO HEK293 cells, seeded at a density of 150,000 cells per well, were transfected with either 20 ng of psiCHECK reporter (as control) or a construct of luciferase with full-length *Ifnb1* 3′UTR (201 nucleotides; psiCHECK-RL-*Ifnb1* 3′UTR reporter) using Lipofectamine 2000. Twenty-four hours after transfection, cells were lysed, followed by dual-luciferase assay (Promega, E1960). RL values were normalized against firefly luciferase (FL) levels for each sample.

For the tether-function assays, we used the λN:BoxB tethering approach (*33*). Briefly, an RL reporter containing five BoxB hairpins in its 3′ UTR, followed by a Poly(A) tail, and a variant form of the reporter, which instead of Poly(A) tail, contains a self-cleaving HhR at the 3′ end (*35*), were used. The reporter was cotransfected with the plasmid encoding a λN peptide–fused protein of interest and a plasmid expressing FL. Twenty-four hours after transfection, luciferase activities were measured using dual-luciferase assay (Promega, E1960). RL values were normalized against FL levels to determine the effect of the tethered protein on the expression of the target mRNA.

### Polysome profiling

Polysome profiling was performed as previously described (*77*) with minor modifications. Briefly, HEK293 cells at 80 to 90% confluency were treated with cycloheximide (CHX; 100 μg/ml) for 5 min at 37°C. Subsequently, cells were washed twice with ice-cold phosphate-buffered saline (PBS) containing CHX (100 μg/ml), suspended in a hypotonic buffer [5 mM tris-HCl (pH 7.4), 1.5 mM KCl, 2.5 mM MgCl_2_, CHX (100 μg/ml), RNase inhibitor (200 μg/ml), and 2 mM dithiothreitol (DTT)], supplemented with EDTA-free protease inhibitor tablet (Roche, 04693124001), and lysed using a detergent mixture (0.5% Triton X-100 and 0.5% sodium deoxycholate). The amount of RNA in the supernatant was quantified using a NanoDrop 2000 spectrophotometer (Thermo Fisher Scientific). Equal amounts of RNA were loaded onto a 10 to 50% sucrose gradient containing 20 mM Hepes-KOH (pH 7.6), 100 mM KCl, 5 mM MgCl_2_, CHX (100 μg/ml), and RNase inhibitor (10 μ/ml), supplemented with EDTA-free protease inhibitor tablet. Gradients were centrifuged at 36,000 rpm for 2 hours at 4°C. The absorbance at 254 nm was measured to analyze mRNA sedimentation across sucrose gradients using TracerDAQ software.

### RNA extraction and reverse transcription polymerase chain reaction

Total RNA from each sample was extracted using easy-BLUE Total RNA Extraction Kit (iNtRON Biotechnology, 17061), following the manufacturer’s instructions. Reverse transcription was performed using oligo-dT (*18*) and SuperScript III (Invitrogen, 18080044), using 1 μg of total RNA. The mRNA levels were analyzed using the CFX Connect Real-Time PCR Detection System (Bio-Rad) with the SensiFAST SYBR No-ROX Kit (Bioline) and gene-specific primers (table S1). Quantification was carried out using the comparative Ct method.

### Immunoprecipitation

Cells were rinsed with cold PBS and harvested by scraping into lysis buffer [40 mM Hepes (pH 7.5), 120 mM NaCl, 1 mM EDTA, 50 mM NaF, and 0.3% CHAPS], supplemented with a complete EDTA-free protease inhibitor tablet (Roche, 04693124001) and phosphatase inhibitor cocktail (Sigma-Aldrich, P5726, P0044). Precleared lysates containing 500 μg of protein were incubated with 1 μg of anti-FLAG, anti-HA, or anti-v5 antibody and 40 μl of Protein G agarose beads slurry (Millipore, 16-266) in the presence of ribonuclease A (Thermo Fisher Scientific, EN0531) per immunoprecipitation (IP) overnight at 4°C. The beads were then washed three times for 10 min each with wash buffer [50 mM Hepes (pH 7.5), 150 mM NaCl, 1 mM EDTA, 50 mM NaF, and 0.3% CHAPS], supplemented with a complete EDTA-free protease inhibitor tablet and phosphatase inhibitor cocktail. Proteins were eluted from the beads in the SDS sample buffer.

### RNA immunoprecipitation

HEK293 cells cultured in two 10-cm plates were transfected with v5-GIGYF1–expressing plasmid, followed by treatment with poly(I:C) (1 μg/ml) or mock for 6 hours. Cells were lysed in lysis buffer A [50 mM Hepes-KOH (pH 7.4), 2 mM EDTA, 10 mM pyro-phosphate, 10 mM beta-glycerophosphate, 40 mM NaCl, 1% Trition X-100, supplemented with a complete EDTA-free protease inhibitor tablet (Roche, 04693124001) and phosphatase inhibitor cocktail (Sigma-Aldrich, P5726, P0044) containing SuperaseIn (40 U/ml)]. Insoluble material was removed by centrifugation at 20,000*g* for 10 min at 4°C. Protein concentration was measured by Bradford assay, and 1 mg of lysate was precleared by incubating with 50 ml of 50% protein G agarose fast flow beads (Millipore, 16-266) for 3 hours at 4°C with gentle agitation. The cleared lysates were collected by centrifugation at 3000*g* for 1 min at 4°C and collecting the supernatant. In parallel, 1 mg of anti-v5 antibody was incubated with 50 ml of 50% protein G agarose fast flow beads for on an end-over-end rotator for 1 hour. at 4°C. For IP, the precleared lysates were incubated with the antibody + bead mixture, in 1-ml total volume on an end-over-end rotator for 2 hours at 4°C. The precipitated beads were then washed three times with 1 ml of buffer A, twice with buffer B [15 mM Hepes-KOH (pH 7.4), 7.5 mM MgCl_2_, 100 mM KCl, 2 mM DTT, and 1.0% Triton X-100], and resuspended in 100 ml of buffer B. The final mix (20 ml) was used for WB and the remaining was used for RNA extraction.

### Proximity ligation assay

PLA was conducted using Duolink reagents (Sigma-Aldrich, DUO92101) in accordance with the manufacturer’s instructions. In brief, cells were fixed with a solution of 4% paraformaldehyde-sucrose for 15 min and permeabilized using PBS containing 0.1% Triton X-100 for 15 min. Following that, the cells were blocked with Duolink blocking solution at 37°C for 1 hour and incubated with primary antibodies overnight at 4°C. Before incubating with the PLA probe, the cells were washed with wash buffer A and then incubated with the probe for 1 hour at 37°C, followed by a 30-min ligation step at 37°C. To amplify the PLA signals, an amplification buffer was applied for 100 min at 37°C. Cells were washed with wash buffer B and mounted onto slide glass for Airyscan microscopic imaging (Zeiss).

### Streptavidin-biotin RNA affinity purification assay

*Ifnb1* mRNA 3′UTR (NM_002176.4) was synthesized using the HiScribe T7 High Yield RNA synthesis kit (NEB, E2040S) through in vitro transcription, incorporating biotinylated uridine triphosphate (Jena Bioscience, NU-821-BIO16). For capped biotinylated 3′UTR, 3′-O-Me-m7G(5′)ppp(5′)G RNA Cap Structure Analog (NEB, S1411L) was also added during this process. Biotinylated 3′UTRs were incubated with cytoplasmic lysates in dialysis buffer [10 mM Hepes (pH 7.4), 90 mM KOAc, 1.5 mM MgOAc, and 2.5 mM DTT] for 30 min at room temperature. In competition experiments, a 10-fold excess of nonbiotinylated *Ifnb1* 3′UTR was also included in the dialysis buffer with the biotinylated *Ifnb1* 3′UTR. After 30 min, the binding mixture was subjected to streptavidin resin (Thermo Fisher Scientific, 20349) adsorption for 16 hours at 4°C on a rotary mixer. Following three washes with a wash buffer (dialysis buffer containing 1% NP-40), proteins were eluted from the resin in the SDS sample buffer and analyzed by SDS–polyacrylamide gel electrophoresis.

### Size exclusion chromatography

HEK293 cells were collected by centrifugation and resuspended in 500 μl of lysis buffer containing 50 mM tris-HCl (pH 7.4), 150 mM NaCl, 1 mM EDTA (pH 8.0), 1% Triton X-100, supplemented with a complete EDTA-free protease inhibitor tablet (Roche, 04693124001) and phosphatase inhibitor cocktail (Sigma-Aldrich, P5726, P0044). The lysate was clarified by centrifugation at 20,000*g* for 5 min at 4°C and filtered on an Ultrafree-MC GV Centrifugal filter (Millipore, UFC30GV0S). Superose 6 Increase HR 10/300 column (Cytiva) was equilibrated with SEC buffer [25 mM Hepes (pH 7.5), 150 mM NaCl, and 0.5 mM Tris(2-carboxyethyl)phosphine (TCEP)] at 0.4 ml/min before a total of 10 mg of protein from HEK293 cell lysate was applied to the column. Fractions of 0.5 ml were collected, of which 50 μl was used for WB analysis.

### Generation of stable cell lines for BioID assay

Flp-In T-REx HEK293 cells were transfected using jetPRIME transfection reagent (Polyplus) according to manufacturer’s specifications. Briefly, cells were seeded in six-well plates with DMEM supplemented with 5% FBS, 5% Cosmic calf serum and Pen/Strep (100 U/ml) and transfected with 100 ng of pcDNA5-BAIT_PROTEIN-BirA*-FLAG and 1 μg of POG44 (Flp recombinase). Transfected cells were passaged into 10-cm plates and 48 hours after transfection treated with hygromycin B (200 μg/ml) for stable selection of integrated cells. Selection media was changed every 2 to 3 days until clear visible colonies were present. Mixed clonal populations were pooled and scaled up into three 15-cm plates (1 to freeze and 2 for BioID).

### BioID; affinity purification and trypsin digestion

For BioID experiments, stable cells were grown to ~75% confluency. Bait expression vectors and biotin were induced simultaneously [tetracycline (1 μg/ml) and 50 μM biotin]. After 24 hours of treatment, cells were rinsed once on the plate with ~20 ml of PBS, then scraped into 1 ml of PBS. Cell pellets were collected by centrifugation (500*g* for 5 min) and stored at -80°C for further processing. Cell pellets were thawed on ice and tared weight calculated. A 4:1 (v/w) ratio of ice-cold lysis buffer was added to the cells [50 mM tris-HCl, (pH 7.5), 150 mM NaCl, 1% NP40, 0.4% SDS, 1.5 mM MgCl_2_, 1 mM EGTA, benzonase, and Sigma-Aldrich protease inhibitors]. Cells were dispersed with a P1000 pipette tip (~10 to 15 aspirations) and subjected to a rapid freeze/thaw cycle (dry ice to 37°C water bath). Lysates were rotated at 4°C for 30 min and then centrifuged at 16,000*g* for 20 min at 4°C. Supernatant was collected (with 20 μl of aliquot saved for WB) into new tubes for affinity purification (AP). Samples were incubated with 20 μl (packed beads) of streptavidin-Sepharose (GE) (equilibrated in lysis buffer) with rotation overnight at 4°C. Beads were collected (500*g* for 2 min), the supernatant discarded, and the beads transferred to new tubes in 500 μl of lysis buffer. Beads were washed once with SDS wash buffer [50 mM tris-HCl, (pH 7.5) and 2% SDS], twice with lysis buffer, and thrice with 50 mM ammonium bicarbonate (pH 8.0) (ABC) (all wash volumes = 500 μl with centrifugations at 500*g* for 30 s). Beads were resuspended in 100 μl of ABC containing 1 μg of sequencing grade trypsin and gently mixed at 37°C for 4 hours. Fresh trypsin (1 μg) was added, and the samples were rotated overnight. Supernatant was collected (500*g* for 2 min) and the beads washed with 100 μl of molecular biology grade H_2_O and pooled with peptides. Digestion was terminated by acidification with formic acid (50 μl of 10% stock = 2% final concentration). Samples were then centrifuged (16,000*g* for 5 min) and ~ 90% of the sample was transferred to a new tube and dried using vacuum centrifugation.

### Mass spectrometry

Each sample (5 μl in 2% formic acid; corresponding to one-eighth of a 15-cm tissue culture dish) was directly loaded at 800 nl/min onto an equilibrated high-performance liquid chromatography column. The peptides were eluted from the column over a 90-min gradient generated by a Eksigent ekspert nanoLC 425 (Eksigent, Dublin CA) nano-pump and analyzed on a TripleTOF 6600 instrument (SCIEX, Concord, Ontario, Canada). The gradient was delivered at 400 nl/min starting from 2% acetonitrile with 0.1% formic acid to 35% acetonitrile with 0.1% formic acid over 90 min followed by a 15-min clean-up at 80% acetonitrile with 0.1% formic acid, and a 15-min equilibration period back to 2% acetonitrile with 0.1% formic acid, for a total of 120 min. To minimize carryover between each sample, the analytical column was washed for 2 hours by running an alternating sawtooth gradient from 35% acetonitrile with 0.1% formic acid to 80% acetonitrile with 0.1% formic acid at a flow rate of 1500 nl/min, holding each gradient concentration for 5 min. Analytical column and instrument performance were verified after each sample by loading 30-fmol bovine serum albumin (BSA) tryptic peptide standard with 60-fmol α-casein tryptic digest and running a short 30-min gradient. Time-of-flight (TOF) mass spectrometry (MS) mass calibration was performed on BSA reference ions before running the next sample to adjust for mass drift and verify peak intensity. Samples were analyzed data-dependent acquisition (DDA) mode. The DDA method consisted of 1 250-ms MS1 TOF survey scan from 400 to 1800 Da followed by 10 100-ms MS2 candidate ion scans from 100 to 1800 Da in high-sensitivity mode. Only ions with a charge of 2+ to 5+ that exceeded a threshold of 300 cps were selected for MS2, and former precursors were excluded for 7 s after one occurrence.

### MS data analysis

All raw (WIFF and WIFF.SCAN) files were saved in the Gingras lab local interaction proteomics LIMS, ProHits (*78*). mzXML files were generated from raw files using the ProteoWizard converter (v3.0.4468) and SCIEX converter (v1.3 beta), implemented within ProHits. The searched database contained the human complement of the RefSeq protein database (version 57) complemented with SV40 large T-antigen sequence (72,226 sequences searched; including reversed sequences). mzXML files were searched by Mascot (v2.3.02) and Comet (v2016.01 rev. 2) using the following parameters: up to two missed trypsin cleavage sites, methionine oxidation and asparagine/glutamine deamidation as variable modifications. The fragment mass tolerance was 0.15 Da, and the mass window for the precursor was ±40 ppm with charges of 2+ to 4+ (both monoisotopic mass). Search engine results were analyzed using the Trans-Proteomic Pipeline (TPP v4.6 OCCUPY rev 3) (*79*) via iProphet (*80*). SAINTexpress (v3.6.3) (*81*) was used to score proximity interactions from DDA data. SAINTexpress calculates, for each prey protein identified by a given bait, the probability of a true proximity interaction relative to negative control runs using spectral counting as a proxy for abundance. Bait runs (two biological replicates for N- and C-terminal tagged GIGYF1 each; four total) were compared against eight negative control runs consisting of four BirA*-FLAG-only samples and four 3xFLAG-only samples, which were compressed to four “virtual controls” to maximize stringency of scoring. Preys with a FDR ≤ 1% (Bayesian estimation based on distribution of the Averaged SAINT scores across both biological replicates) were considered high-confidence proximity interactions.

All MS files used in this study were deposited at MassIVE (http://massive.ucsd.edu) and have been assigned the following accession number: MSV000093103. The ProteomeXchange accession is PXD046126. Gene Ontology analyses were performed using the online g:profiler tool: https://biit.cs.ut.ee/gprofiler/gost.

### Statistical analysis

Statistical tests were performed using Prism 6 (GraphPad). Error bars represent SD from the mean of independent replicates. Number of replicates used in each analysis is indicated in the corresponding figure legend. *P* < 0.05 were considered significant.
