# Supplementary material for: Repression of mRNA translation initiation by GIGYF1 via disrupting the eIF3-eIF4G1 interaction
Source: Sci Adv. 2024 Jul 17;10(29):eadl5638. doi: 10.1126/sciadv.adl5638 (PMC466957; doi:10.1126/sciadv.adl5638)
Supplement: Supplementary file 1 — Figs. S1 to S8 Table S1 Legends for data S1 and S2 [file sciadv.adl5638_sm.pdf]

Science Advances
AAAS

Supplementary Materials for

Repression of mRNA translation initiation by GIGYF1 via disrupting the eIF3-eIF4G1 interaction

Jung-Hyun Choi *et al.*

Corresponding author: Xu Zhang, zx@ism.cams.cn; Nahum Sonenberg, nahum.sonenberg@mcgill.ca; Seyed Mehdi Jafarnejad, sm.jafarnejad@qub.ac.uk

Sci. Adv. **10**, eadl5638 (2024)
DOI: 10.1126/sciadv.adl5638

The PDF file includes:

Figs. S1 to S8
Table S1
Legends for data S1 and S2

Other Supplementary Material for this manuscript includes the following:

Data S1 and S2

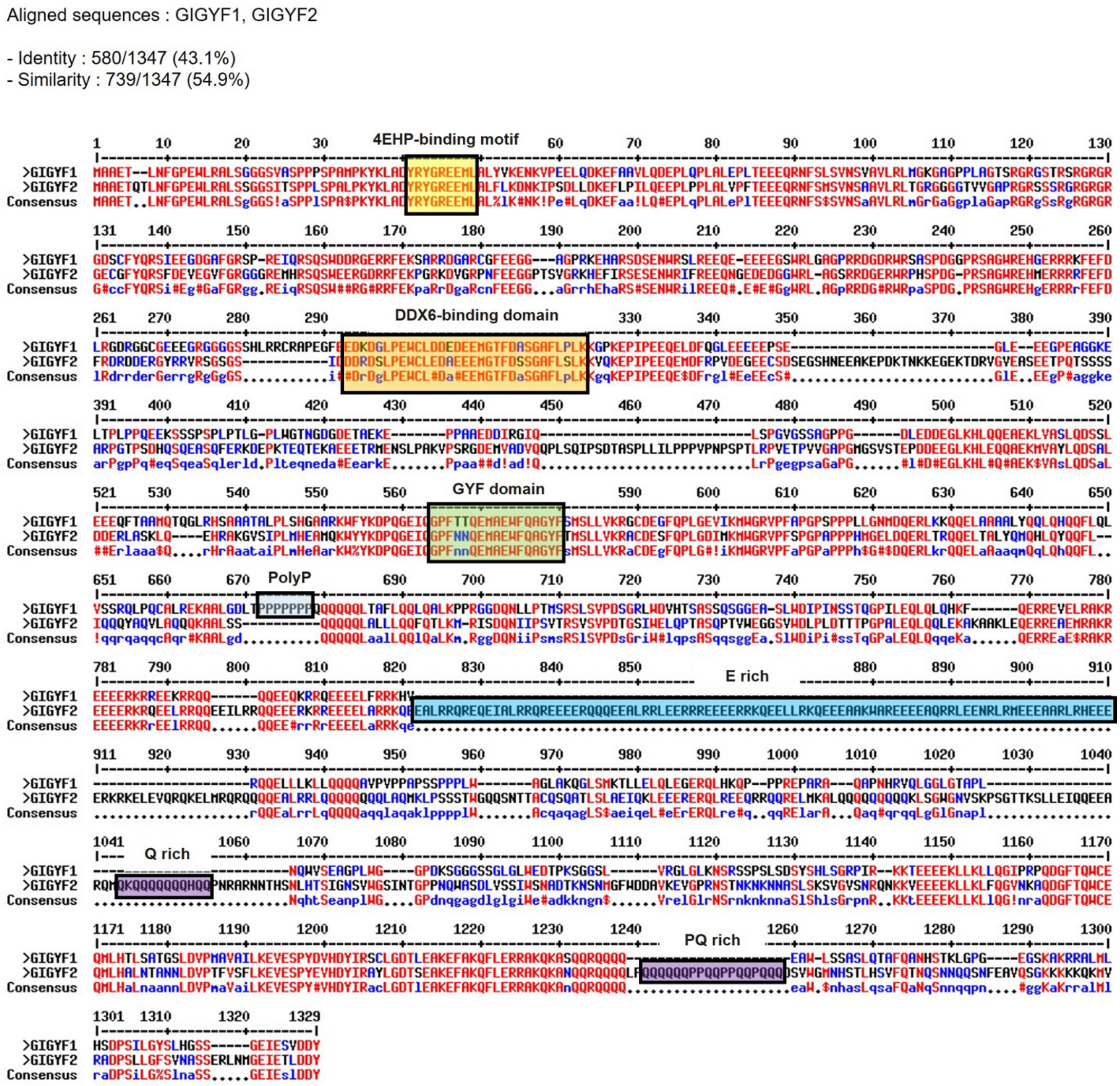

Fig. S1.

Alignment of the human GIGYF1 and GIGYF2 protein sequences; related to Figure 1. Alignment of the human GIGYF1 (NP_001362694.1) and GIGYF2 (NP_001096616.1) protein sequences by multAlin software (http://multalin.toulouse.inra.fr/multalin/multalin.html).

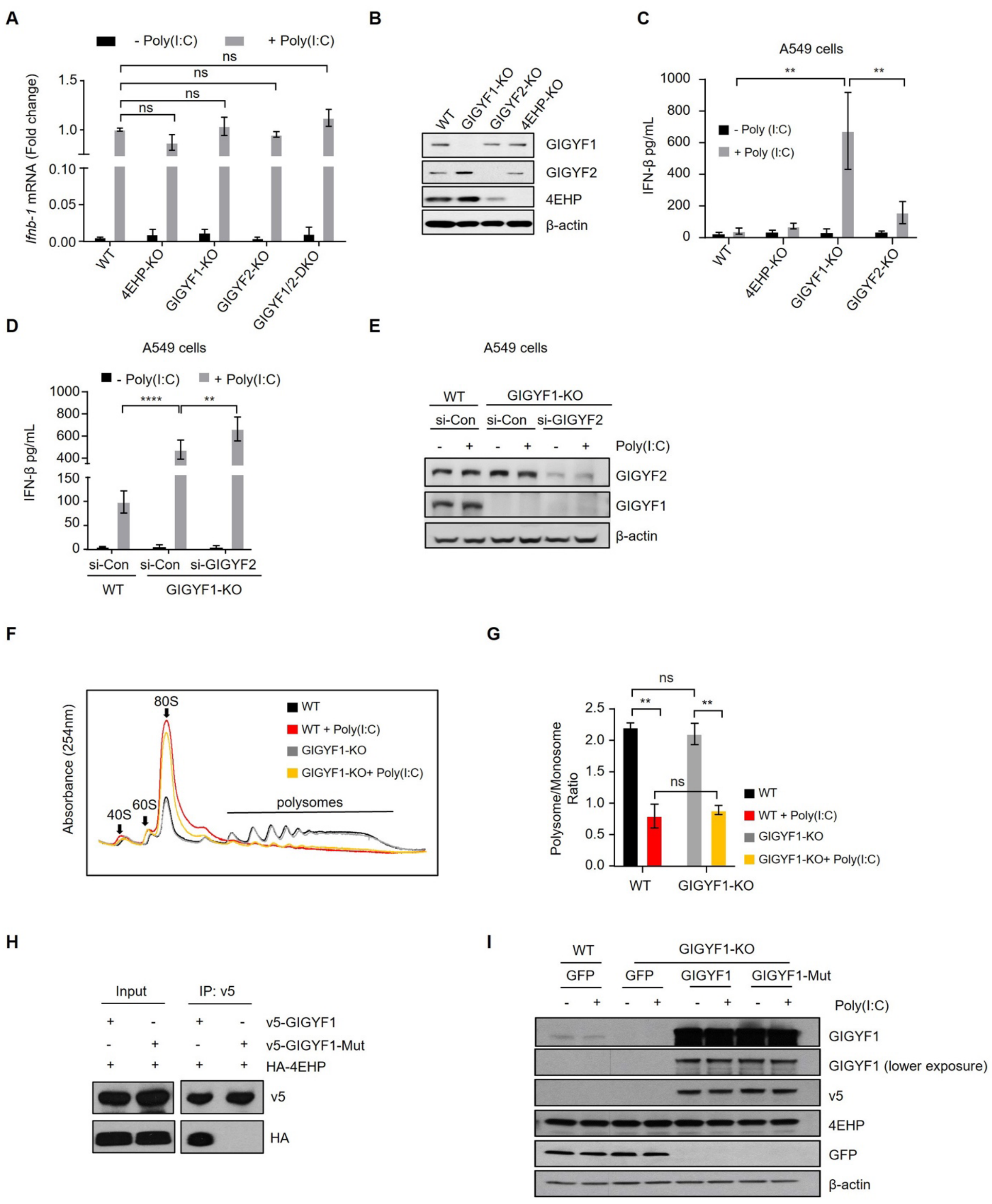

Fig. S2.

Translational repression of target mRNAs by GIGYF1; related to Figure 1. (**A**) RT-qPCR analyses of samples described in Figure 1D. *GAPDH* mRNA level was used as a control for

normalization. Data are presented as mean ± SD (n=3). ns = non-significant, analyzed by one-way ANOVA with Bonferroni's post-hoc test. (**B**) Western blot analysis with the indicated antibodies of WT, 4EHP-KO, GIGYF1-KO, and GIGYF2-KO A549 cell lysates. (**C**) ELISA measurement of IFN-β production in WT, 4EHP-KO, GIGYF1-KO, and GIGYF2-KO A549 cells following 6 h of treatment with 1 μg/mL poly(I:C). Data are presented as mean ± SD (n=3). **P < 0.01; one-way ANOVA with Bonferroni's post-hoc test. (**D**) ELISA measurement of IFN-β production in WT and GIGYF1-KO A549 cells transfected with a control siRNA (si-Con) or siRNA against GIGYF2 (si-GIGYF2) following 6 h of treatment with 1 μg/mL poly(I:C). Data are presented as mean ± SD (n=3). **P < 0.01, ****P < 0.0001; one-way ANOVA with Bonferroni's post-hoc test. (**E**) Western blot analysis of cell lysates from (D). (**F**) Polysome profiling using WT and GIGYF1-KO HEK293 cells with or without poly(I:C) treatment. 1 μg/mL poly(I:C) was used to stimulate the cells for 6 h. (**G**) Polysome (fractions 6-12) / monosome (fractions 4-5) (P/M) ratio of samples described in F. Data are presented as mean ± SD. ns = non-significant, **P < 0.01; two-way ANOVA with Bonferroni's post-hoc test. (**H**) Co-immunoprecipitation assay for detection of interactions between GIGYF1 and 4EHP. HA-4EHP and v5-GIGYF1 or the 4EHP-binding (Y39A, Y41A, M46A, L47A) mutant v5-GIGYF1 (v5-GIGYF1 Mut) were co-expressed in HEK293 cells. Cell lysates were immunoprecipitated with anti-v5 antibody. Immunoblotting was performed with the indicated antibodies. Inputs represent 2% of the total lysate used in the IP assay. (**I**) Western blot analysis with the indicated antibodies of cell lysates described in Fig. 1J.

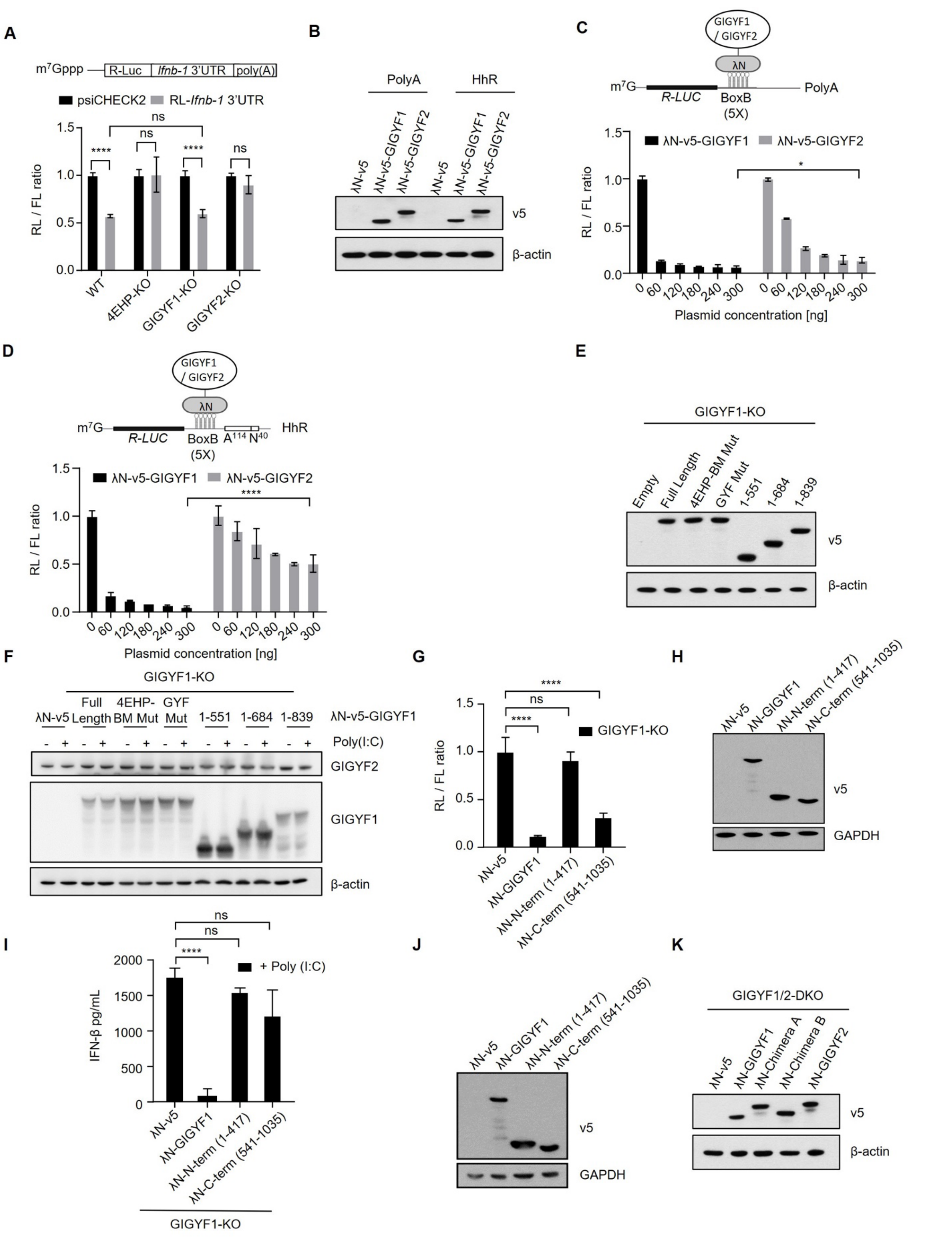

Fig. S3. Robust translational repression of target mRNAs by GIGYF1, compared with GIGYF2; related to Figure 2. (**A**) WT, 4EHP-KO, GIGYF1-KO, and GIGYF2-KO HEK293

cells were transfected with psiCHECK2-RL-*Ifnb1* 3′ UTR reporter or the psiCHECK2 reporter (as control). RL and FL activities were measured 24 h after transfection. The RL/FL ratio in psiCHECK2-RL-*Ifnb1* 3′ UTR reporter–expressing cells was normalized to the psiCHECK2-expressing cells. Data are presented as mean ± SD (n=3). ns = non-significant, ****P < 0.0001; two-way ANOVA with Bonferroni's post-hoc test. (**B**) Western blot analysis of cell lysate from Figure 2C with the indicated antibodies. (**C** & **D**) Tether-function assays for measurement of repression of the deadenylation-permissive RL-5boxB-polyA (**C**) or deadenylation-resistant RL-5BoxB-HhR reporters (**D**) upon coexpression with increasing amounts of λN-v5-GIGYF1 or λN-v5-GIGYF2 plasmids in HEK293 cells. Data are presented as mean ± SD (n=3). *P < 0.05, ****P < 0.0001; two-way ANOVA with Bonferroni's post-hoc test. (**E-F**) Western blotting with the indicated antibodies using lysates from cells shown in Figure 2E and 2F, respectively. (**G**) Tether-function assay with the full-length, N-terminal (aa1-417), and C-terminal (aa541-1035) fragments of GIGYF1 in GIGYF1-KO HEK293 cells. Cells were co-transfected with the indicated plasmid along with the deadenylation-resistant RL-5BoxB-HhR and FL control plasmid, followed by dual-luciferase measurement assay 24 h post-transfection. Data are presented as mean ± SD (n=3). ns = non-significant, ****P < 0.0001; one-way ANOVA with Bonferroni's post-hoc test. (**H**) Western blotting with the indicated antibodies using lysates from cells shown in (G). (**I**) ELISA measurement of IFN-β production in GIGYF1-KO HEK293 cells overexpressing the full-length GIGYF1 and the indicated truncated isoforms following 6 h of treatment with 1 μg/mL poly(I:C). Data are presented as mean ± SD (n=3). ns = non-significant, ****P < 0.0001; one-way ANOVA with Bonferroni's post-hoc test. (**J**) Western blotting with the indicated antibodies using lysates from cells shown in (I). (**K**) Western blotting with the indicated antibodies using lysates from cells shown in Figure 2H.

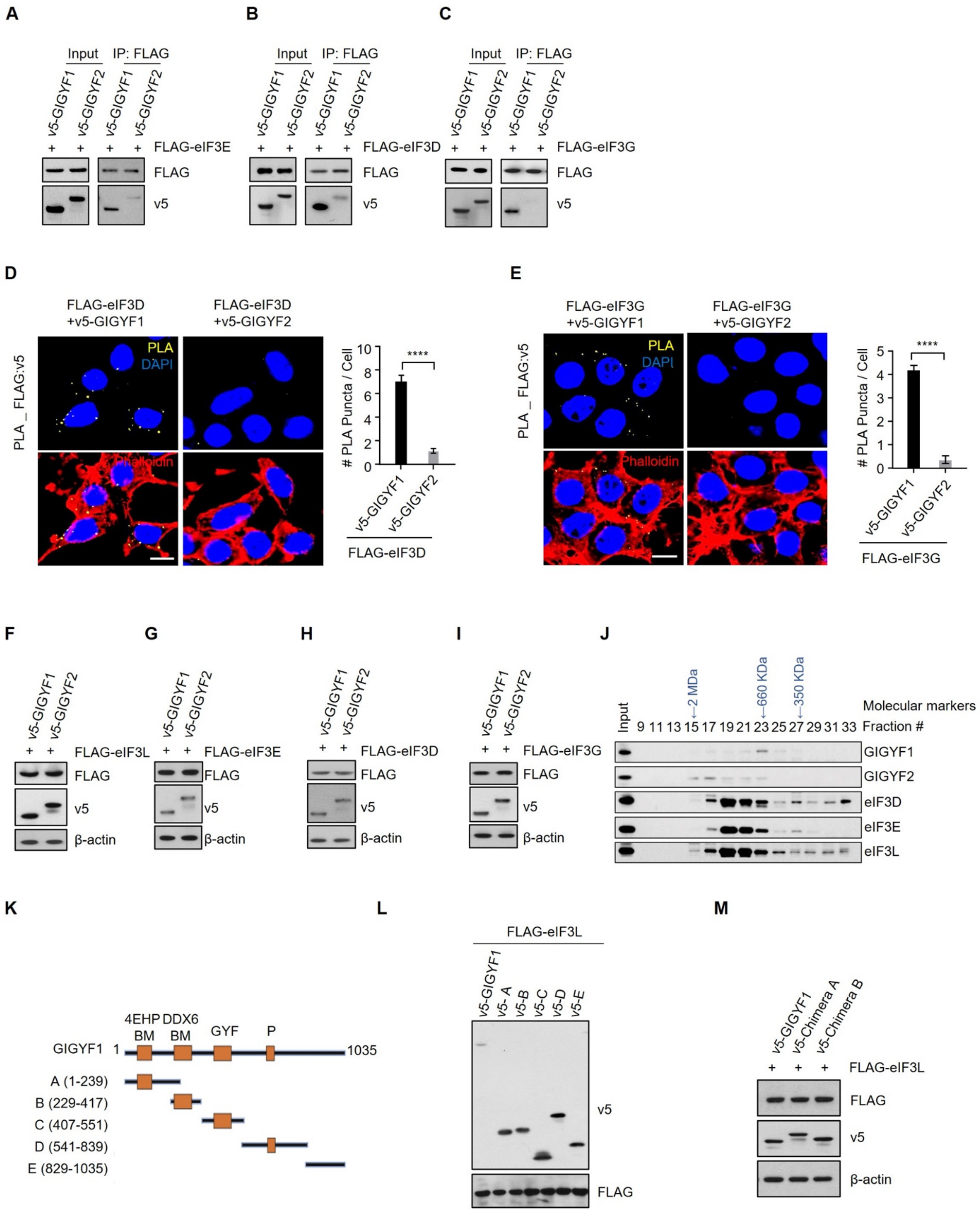

Fig. S4.

Interactions between GIGYF1 and eIF3 subunits; related to Figure 3. (**A-C**) Co-IP assay for detecting the interaction of FLAG-eIF3E (A), FLAG-eIF3D (B), or FLAG-eIF3G (C) with v5-GIGYF1 or v5-GIGYF2 in HEK293T cells. Whole-cell lysates were prepared 24 h after

transfection and subjected to immunoprecipitation using an anti-FLAG antibody followed by blotting with the indicated antibodies. (**D**) *Left*: PLA for detection of GIGYF1-eIF3D interaction in HEK293T cells transfected with vectors expressing v5-GIGYF1 or v5-GIGYF2 along with FLAG-eIF3D. 24 h post-transfection cells were fixed and subjected to PLA using FLAG and v5 antibodies. Scale bar = 10 µm. *Right*: The bar graphs represent the number of PLA signals from at least 30 cells, counted in each sample. n=5 independent experiments. Data are presented as mean ± SD. ns= non-significant, ****P< 0.0001; unpaired t-test. (**E**) *Left*: PLA for detection of GIGYF1-eIF3G interaction in HEK293T cells transfected with vectors expressing v5-GIGYF1 or v5-GIGYF2 along with FLAG-eIF3G. 24 h post-transfection cells were fixed and subjected to PLA using FLAG and v5 antibodies. Scale bar = 10 µm. *Right*: The bar graphs represent the number of PLA signals from at least 30 cells, counted in each sample. n=5 independent experiments. Data are presented as mean ± SD. ns= non-significant, ****P< 0.0001; unpaired t-test. (**F-I**) Western blot analysis of the samples in Figure 3B, Figure 3C, Supp. Figure 4D, and Supp. Figure 4E respectively with the indicated antibodies. (**J**) Fractionation of endogenous GIGYF1, GIGYF2, and the indicated subunits of eIF3 complex by size-exclusion chromatography. A total of 10 mg of proteins from HEK293 cells was loaded onto a Superose 6 column and ran at a flow rate of 0.4 mL/min. Fractions of 0.5 mL were collected, and 50 µL of each fraction was subjected to western blot analysis. The elution position of the molecular size markers is shown. (**K**) Schematic of the domain structures of full-length (FL) GIGYF1 and truncated isoforms A-E used in Figure 3D and Supp. Figure 5A. (**L**) Western blot analysis of the samples in Figure 3D with the indicated antibodies. (**M**) Western blot analysis of the samples in Figure 3E with the indicated antibodies.

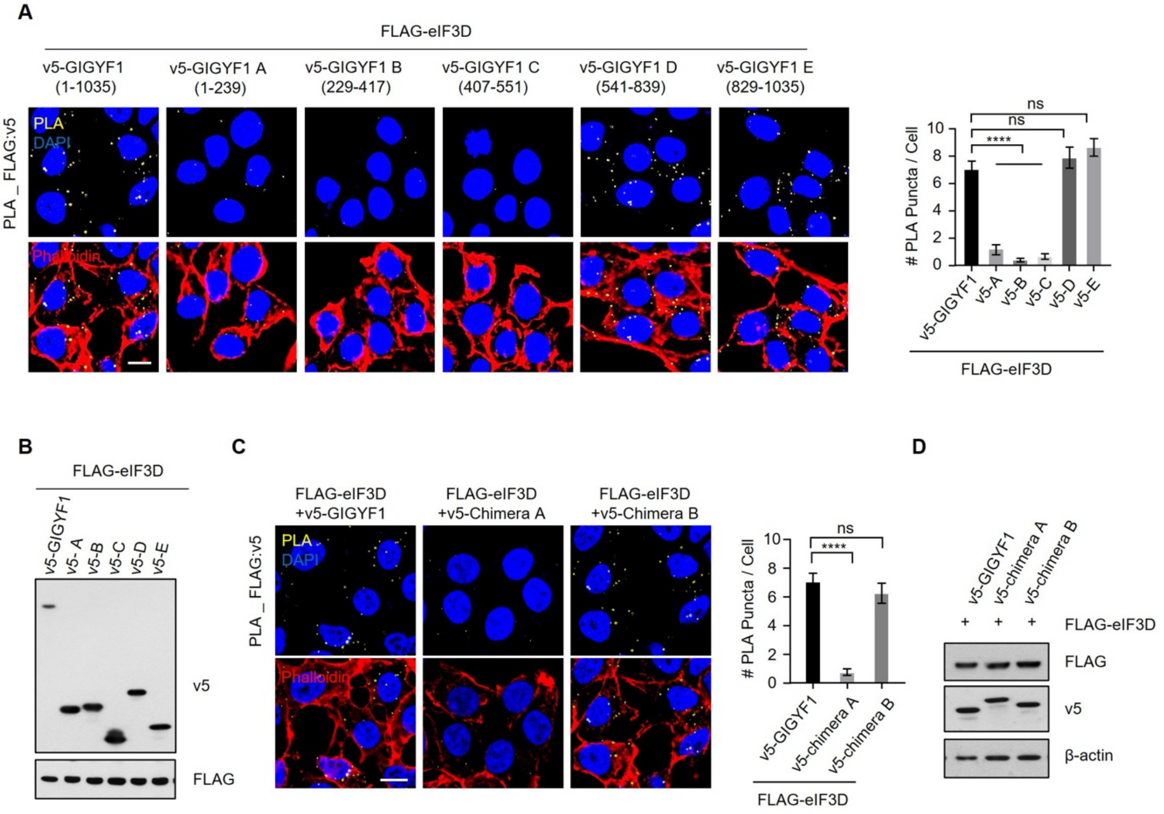

Fig. S5.

The C-terminal region of GIGYF1 mediates its interactions with eIF3D; related to Figure 3. (**A**) *Left*: HEK293T cells were co-transfected with FLAG-eIF3D and the full-length or indicated GIGYF1 truncation mutants (see Supp. Fig. 4K for more details) or full-length GIGYF1 (as control). 24 h post-transfection cells were fixed and subjected to PLA using FLAG and v5 antibodies. PLA signals are shown in yellow. The nucleus and actin cytoskeleton were counterstained with DAPI and phalloidin (red), respectively. Scale bar = 10 µm. *Right*: The bar graphs represent the number of PLA signals from at least 20 cells, counted in each sample. n=3 independent experiments. ns = non-significant, ****P< 0.0001; one-way ANOVA with Bonferroni's post-hoc test. (**B**) Western blot analysis of cell lysates from (A) with the indicated antibodies. (**C**) *Left*: PLA assay for detection of the interactions between eIF3D and the indicated two chimeric constructs described in Figure 2G. *Right*: The bar graphs represent the number of PLA signals from at least 20 cells, counted in each sample. n=3 independent experiments. Data are presented as mean ± SD. ns= non-significant, ****P < 0.0001; one-way ANOVA with Bonferroni's post-hoc test. (**D**) Western blot analysis of cell lysates from (C) with the indicated antibodies.

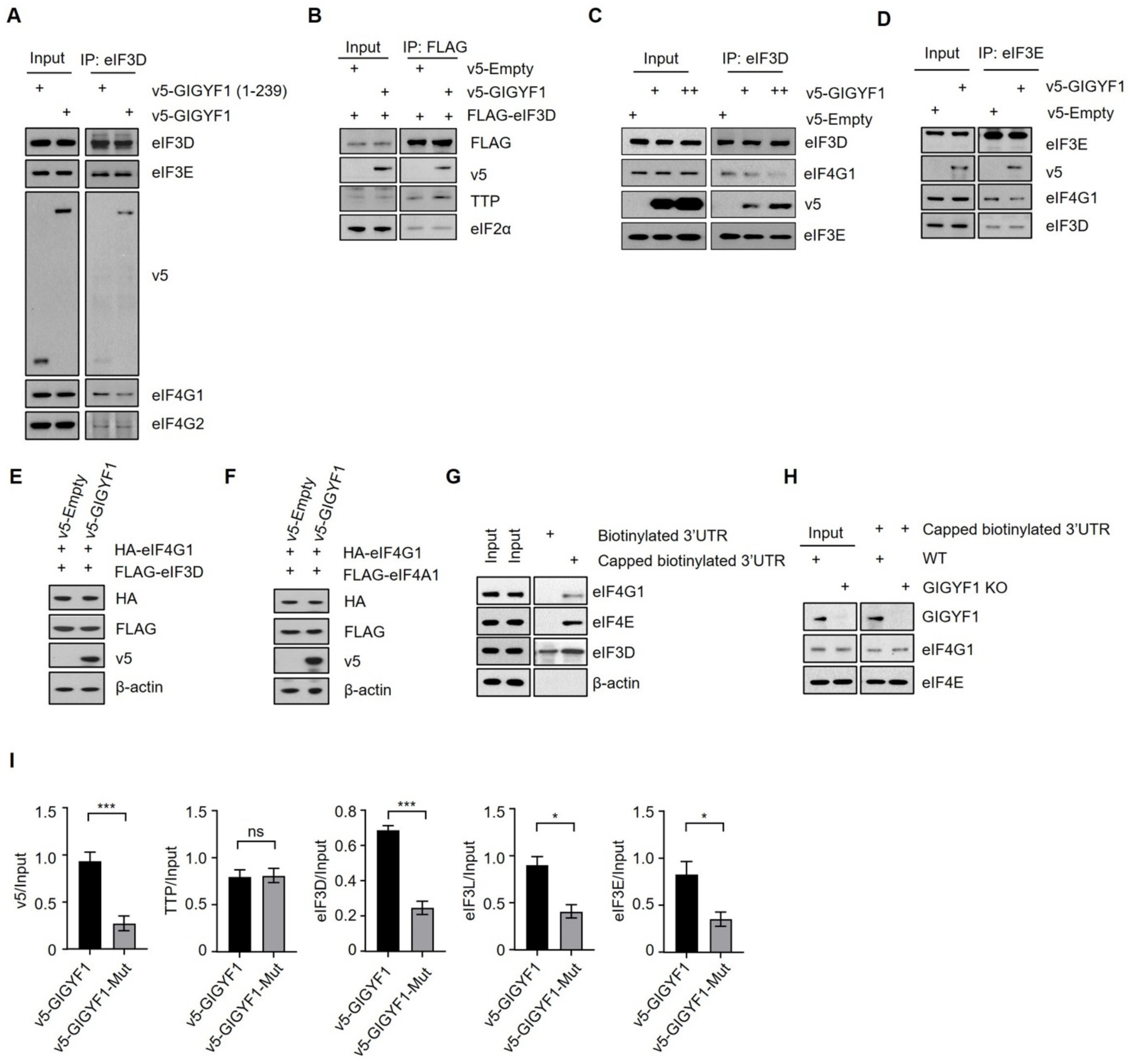

Fig. S6.

Ectopic expression of GIGYF1 reduces interactions of endogenous eIF3 and eIF4G1 proteins; related to Figure 4. (**A**) Co-IP assay for assessing the impact of GIGYF1 on interaction of eIF3D with eIF3E, eIF4G1, and eIF4G2. GIGYF1-KO HEK293 cells were transfected with vectors expressing the v5-tagged full-length or N-terminal fragment (aa1-239) of GIGYF1. Whole-cell lysates were prepared 24 h after transfection and subjected to immunoprecipitation using eIF3D antibody followed by blotting with the indicated antibodies. (**B**) Co-IP assay for detection of interactions of FLAG-eIF3D with eIF2α and TTP in GIGYF1-KO cells expressing v5-GIGYF1 or v5-Empty control plasmids. Whole-cell lysates were prepared 24 h after transfection and subjected to immunoprecipitation using FLAG antibody followed by blotting with the indicated antibodies. (**C**) Co-IP assay for detection of interactions of eIF3D with eIF3E and eIF4G1 in GIGYF1-KO cells expressing increasing amounts of GIGYF1. Whole-cell lysates were prepared 24 h after transfection and subjected to immunoprecipitation using anti-eIF3D antibody followed by blotting with the indicated antibodies. (**D**) Co-IP assay for detection of interactions of

eIF3E with eIF3D and eIF4G1 in GIGYF1-KO cells expressing v5-Empty control or v5-GIGYF1. (**E-F**) Western blot analysis of expression of the indicated protein in samples from Figure 4C and 4D, respectively. (**G**) Streptavidin-biotin RNA affinity purification assay with capped or uncapped biotinylated *Ifnb1* 3′ UTR. The pulled-down proteins were subjected to western blotting and probed with the indicated antibodies. (**H**) Capped biotinylated *Ifnb1* 3′ UTR was incubated with lysates from WT and GIGYF1-KO cells. The pulled-down proteins were subjected to western blotting and probed with the indicated antibodies. (**I**) Quantification of the pulled-down proteins in Figure 4G. The co-purified proteins were normalized to the corresponding input. n=3 independent experiments. *P< 0.05, ***P< 0.001; unpaired t-test.

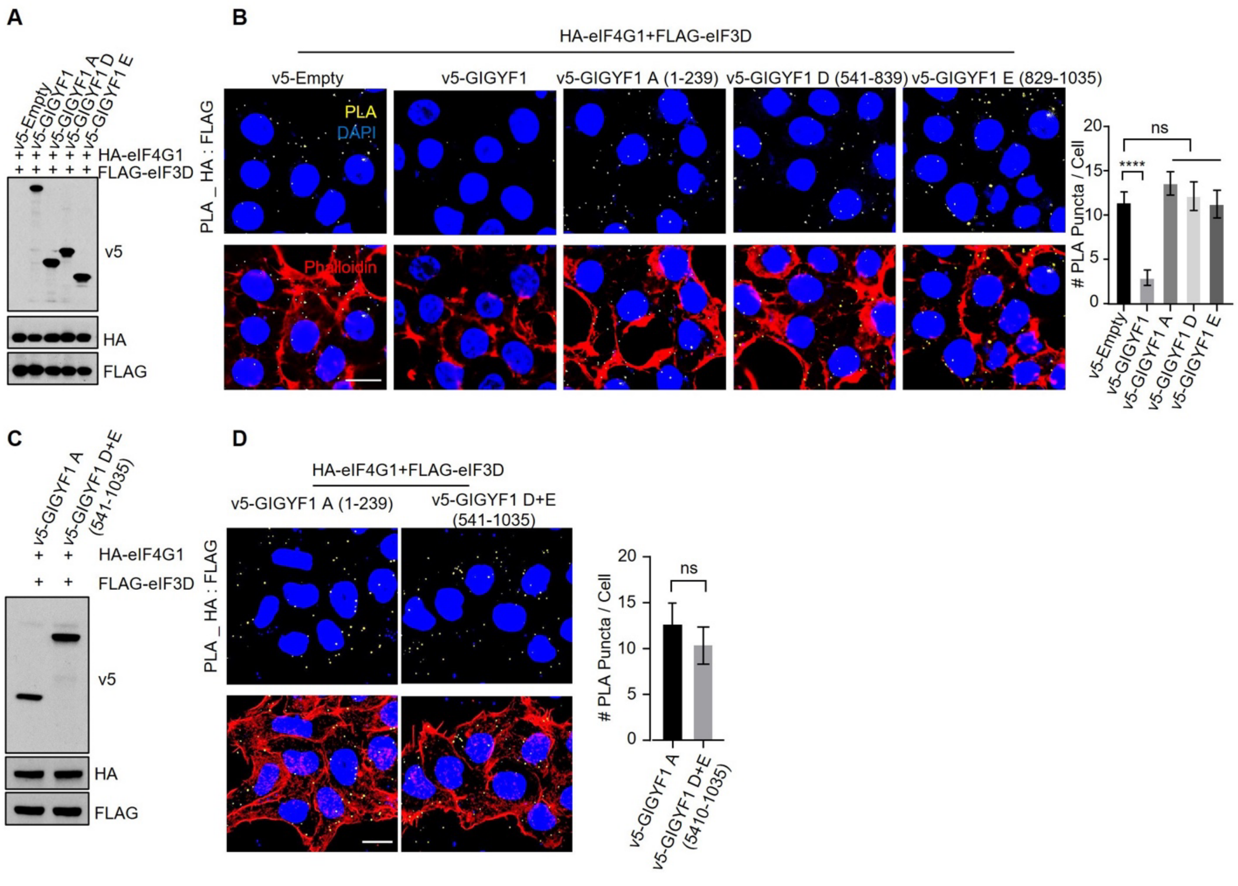

Fig. S7.

C-terminal truncated isoforms of GIGYF1 do not affect eIF4G1-eIF3D interaction; related to Figure 4. (**A**) Western blot analysis of cell lysates from (B). (**B**) PLA assay for detection of HA-eIF4G1 and FLAG-eIF3D in GIGYF1-KO cells transfected with v5-GIGYF1 or truncated isoforms of GIGYF1 (aa1-239, aa541-839 and aa829-1035). (**C**) Western blot analysis of cell lysates from (D). (**D**) PLA assay for detection of HA-eIF4G1 and FLAG-eIF3D in GIGYF1-KO cells transfected with aa1-239 or aa541-1035 truncated isoforms of GIGYF1. 24 h post-transfection cells were fixed and subjected to PLA using HA and FLAG antibodies. The bar graphs represent the number of PLA signals from at least 30 cells, counted in each sample. n=3 independent experiments. ****P< 0.0001; unpaired t-test.

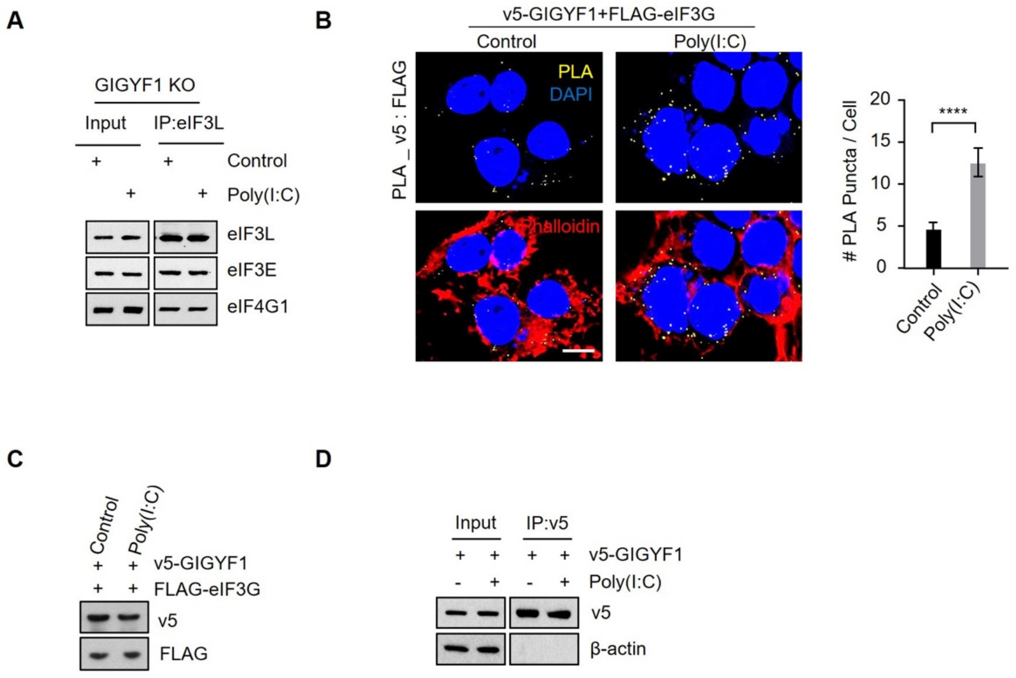

Fig. S8.

GIGYF1 abrogates eIF3/eIF4G1 interaction upon poly(I:C) stimulation; related to Figure 5. (**A**) Co-IP assay to detect the interaction of endogenous eIF3L, eIF3E, and eIF4G1 upon poly(I:C) stimulation in GIGYF1-KO cells. Whole-cell lysates were prepared 24 h after transfection and subjected to immunoprecipitation using anti-eIF3L antibody followed by blotting with the indicated antibodies. (**B**) *Left*: PLA assay for detection of interactions between v5-GIGYF1 and FLAG-eIF3G upon poly(I:C) stimulation. PLA signals are shown in yellow, the nucleus and Actin cytoskeleton were counterstained with DAPI and phalloidin (red), respectively. Scale bar = 10 µm. *Right*: The bar graphs represent the number of PLA signals from at least 20 cells, counted in each sample. n=3 independent experiments. Data are presented as mean ± SD. ns= non-significant, ****P< 0.0001; unpaired t-test. (**C**) Western blot analysis of cell lysates from (B). (**D**) Western blot analysis of the indicated protein in the v5-GIGYF1 RIP assay (Figures 5I). v5-GIGYF1 was immunoprecipitated using a v5 antibody in vehicle and poly(I:C)-treated HEK293 cells. Precipitated proteins were separated by SDS-PAGE and probed with the specified antibodies.

Name	Sequence (5' to 3')	Application
**IFN-β-Fwd**	AAACTCATGAGCAGTCTGCA	RT-PCR
**IFN-β-Rv**	AGGAGATCTTCAGTTTCGGAGG	RT-PCR
**GAPDH-Fwd**	TGGGTGTGAACCATGAGAAG	RT-PCR
**GAPDH-Rv**	ATGGACTGTGGTCATGAGTC	RT-PCR
**GIGYF1 (4EHP BM Mut)-Fwd**	CTGACGCCCGTGCTGGGCGAGAGGAAGCGGCGGCTCTC	Cloning
**GIGYF1 (4EHP BM Mut)-Rv**	GAGAGCCGCCGCTTCCTCTCGCCCAGCACGGGCGTCAG	Cloning
**GIGYF1 (GYF Mut)-Fwd**	CACCAGCAGTGACATGGAAGCGGCGGCGGCCTGGAACCACTCTGC	Cloning
**GIGYF1 (GYF Mut)-Rv**	GCAGAGTGGTTCCAGGCCGCCGCCGCTTCCATGTCACTGCTGGTG	Cloning
**GIGYF1(1-551)-Fwd**	GAGCTCGAGATGGCAGCAGAGACACTCAA	Cloning
**GIGYF1(1-551)-RV**	GAGGCGGCCGCCTACAGCCGCTCCTGGTCCATG	Cloning
**GIGYF1(1-684)-Fwd**	GAGCTCGAGATGGCAGCAGAGACACTCAA	Cloning
**GIGYF1(1-684)-RV**	GAGGCGGCCGCCTATTTATGTTGCAGCTGGAGT	Cloning
**GIGYF1(1-839)-Fwd**	GAGCTCGAGATGGCAGCAGAGACACTCAA	Cloning
**GIGYF1(1-839)-RV**	GAGGCGGCCGCCTACCCCAGGCCGCTGCTGCCG	Cloning
**GIGYF1(1-239)-Fwd**	GAGCTCGAGATGGCAGCAGAGACACTCAA	Cloning
**GIGYF1(1-239)-RV**	GAGGCGGCCGCCTAGCCAGCAGAGCGGGGACCA	Cloning
**GIGYF1(229-417)-Fwd**	GAGCTCGAGATGGCCAGCCCTGATGGTGGTCCCCG	Cloning
**GIGYF1(229-417)-RV**	GAGGCGGCCGCCTATCCGGGTGGGCCAGCAGAGGAGC	Cloning
**GIGYF1(407-551)-Fwd**	GAGCTCGAGATGCCCGGGGTGGGCTCCTCTGC	Cloning
**GIGYF1(407-551)-RV**	GAGGCGGCCGCCTACAGCCGCTCCTGGTCCATGTTTC	Cloning
**GIGYF1(541-839)-Fwd**	GAGCTCGAGATG CCACTGCTGGGAAACATGGAC	Cloning
**GIGYF1(541-839)-RV**	GAGGCGGCCGCCTACCCCAGGCCGCTGCTGCCG	Cloning
**GIGYF1(829-1035)-Fwd**	GAGCTCGAGATG GACAAGAGTGGGGGCGGCAG	Cloning
**GIGYF1(829-1035)-RV**	GAGGCGGCCGCCTAGTAGTCATCCACGCTCTCGAT	Cloning
**GIGYF1 sgRNA#1-Fwd**	CACCGTGACTACCGTTATGGGCGAG	CRISPR
**GIGYF1 sgRNA#1-Rv**	AAACCTCGCCCATAACGGTAGTCAC	CRISPR
**GIGYF1 sgRNA#2-Fwd**	CACC AGCTGGCTGACTACCGTTAT	CRISPR
**GIGYF1 sgRNA#2-Rv**	AAACATAACGGTAGTCAGCCAGCTC	CRISPR
**GIGYF1 sgRNA#3-Fwd**	CACCGAAGCTGGCTGACTACCGTTA	CRISPR
**GIGYF1 sgRNA#3-Rv**	AAACTAACGGTAGTCAGCCAGCTTC	CRISPR

Table S1.

List of primers and sgRNAs used in this study.

Data S1. Combined data from two independent BioID experiments using N- or C-terminal tagged BirA*-GIGYF1 in HEK293 cells. N and C (N- and C-terminal, respectively) indicate the location of BirA* fusion protein in relation to the bait protein.

Data S2. Gene Ontology analysis of the BirA*-GIGYF1 proximal proteins identified with BioID assay in HEK293 cells. Gene Ontology analysis of the significant proximity interactors (FDR<0.01) was performed using the online g:profiler tool: https://biit.cs.ut.ee/gprofiler/gost.
